# Microbiota members from body sites of dairy cows are largely shared within individual hosts throughout lactation but sharing is limited in the herd

**DOI:** 10.1186/s42523-023-00252-w

**Published:** 2023-06-12

**Authors:** Mahendra Mariadassou, Laurent X. Nouvel, Fabienne Constant, Diego P. Morgavi, Lucie Rault, Sarah Barbey, Emmanuelle Helloin, Olivier Rué, Sophie Schbath, Frederic Launay, Olivier Sandra, Rachel Lefebvre, Yves Le Loir, Pierre Germon, Christine Citti, Sergine Even

**Affiliations:** 1 Université Paris-Saclay, INRAE, MaIAGE, Jouy-en-Josas, France; 2 IHAP, Université de Toulouse, INRAE, ENVT, Toulouse, France; 3 Ecole Nationale Vétérinaire d’Alfort, Université Paris-Saclay, UVSQ, INRAE, BREED, Maisons-Alfort, France; 4 Université Clermont Auvergne, INRAE, VetAgro Sup, UMR Herbivores, Saint-Genes-Champanelle, France; 5 INRAE, Institut Agro, UMR1253 STLO, Rennes, France; 6 INRAE, UE326 Unité Expérimentale du Pin, Gouffern en Auge, France; 7 INRAE, Université de Tours, UMR ISP, 37380 Nouzilly, France; 8 Université Paris-Saclay, INRAE, BioinfOmics, MIGALE Bioinformatics Facility, Jouy-en-Josas, France; 9 Université Paris-Saclay, UVSQ, INRAE, BREED, Jouy-en-Josas, France; 10 Université Paris-Saclay, INRAE, AgroParisTech, GABI, Jouy‐en‐Josas, France

**Keywords:** Bovine holobiont, Dairy cow microbiota, Vaginal microbiota, Milk microbiota, Nasal microbiota, Oral microbiota, Mastitis susceptibility, Microbial sharing

## Abstract

**Background:**

Host-associated microbes are major determinants of the host phenotypes. In the present study, we used dairy cows with different scores of susceptibility to mastitis with the aim to explore the relationships between microbiota composition and different factors in various body sites throughout lactation as well as the intra- and inter-animal microbial sharing.

**Results:**

Microbiotas from the mouth, nose, vagina and milk of 45 lactating dairy cows were characterized by metataxonomics at four time points during the first lactation, from 1-week pre-partum to 7 months post-partum. Each site harbored a specific community that changed with time, likely reflecting physiological changes in the transition period and changes in diet and housing. Importantly, we found a significant number of microbes shared among different anatomical sites within each animal. This was between nearby anatomic sites, with up to 32% of the total number of Amplicon Sequence Variants (ASVs) of the oral microbiota shared with the nasal microbiota but also between distant ones (e.g. milk with nasal and vaginal microbiotas). In contrast, the share of microbes between animals was limited (< 7% of ASVs shared by more than 50% of the herd for a given site and time point). The latter widely shared ASVs were mainly found in the oral and nasal microbiotas. These results thus indicate that despite a common environment and diet, each animal hosted a specific set of bacteria, supporting a tight interplay between each animal and its microbiota. The score of susceptibility to mastitis was slightly but significantly related to the microbiota associated to milk suggesting a link between host genetics and microbiota.

**Conclusions:**

This work highlights an important sharing of microbes between relevant microbiotas involved in health and production at the animal level, whereas the presence of common microbes was limited between animals of the herd. This suggests a host regulation of body-associated microbiotas that seems to be differently expressed depending on the body site, as suggested by changes in the milk microbiota that were associated to genotypes of susceptibility to mastitis.

**Supplementary Information:**

The online version contains supplementary material available at 10.1186/s42523-023-00252-w.

## Background

Most studies assessing the host-microbe relationship in ruminants focus on local interactions between an organ and its microbiota in relation to specific phenotypes and have not taken into consideration possible distant interactions. As an illustration, studies on the digestive microbiota explore its relationship to feed utilization and performance, studies on milk microbiota deal with mammary health and mastitis, or studies on the vaginal microbiota are interested in metritis [1–7]. Likewise, studies on the effect of host genetics on the microbiota composition mainly focused on specific phenotypes and body-site microbiotas [8–10].

There are studies in which the microbiota of several body sites was characterized simultaneously, providing information on the development of microbiotas associated with different parts of the gastrointestinal tract [11] or the respiratory tract [12], evaluating the contribution of the vertical transfer of microbiota from the dam and the microbiota assembly in calves [13–15], or exploring the impact of nutrition during gestation on the microbiota of heifers and their offspring [16]. Most of these studies pointed out differences in composition and richness of microbiota associated with different anatomic sites but only few explored the microbial interplay between these sites within an animal and between these microbiotas and host genetics. Besides, the temporal dynamics of these microbiotas has been poorly investigated with studies focusing on changes for specific body sites [15, 17–20], as mentioned above.

The present study aims to provide new insights into the bovine holobiont through a more holistic approach. We investigated the dynamics of dairy cow microbiota associated to different body sites throughout the peripartum period and the first lactation, with the objectives to identify the relationships between different factors and the composition of microbial communities and to investigate microbes shared between different body sites within an animal and between animals within a herd. The microbiota (bacterial moiety only) associated with four different anatomical sites—the mouth, nose, vagina and milk—was characterized. As mentioned above, several studies pointed out a relationship between host genetics and its microbiota. The optimization of milk production by the cattle industry has included the genetic selection of some breeds genetically more resistant to mastitis. To take into account the potential effect of this selection process on the structure and dynamics of microbiotas, we used Prim’Holstein cows selected to present different susceptibility to mastitis [21].

A better understanding of the variability of microbiotas between different body sites of the cow, its dynamics throughout lactation and of the microbial sharing between body sites could contribute to a better monitoring of health and performances through the identification of local or remote-site marker taxa or keystone species. The identification of the main factors influencing microbiota composition could also help to propose strategies to modulate the microbiota towards communities that could be beneficial to health or performance. This longitudinal study sheds new light on the dairy cow microbiota throughout lactation and on the sharing of microbes across anatomical sites within and between animals.

## Methods

### Experimental design

The present study has been carried out at the INRAE “Domaine Expérimental du Pin” (https://doi.org/10.15454/1.5483257052131956E12). This dairy cow experimental facility is located in Normandy (France) and approved by national legislation on animal care (French Ministry of Agriculture certification no. D 61-157-001). All experiments were performed in accordance with relevant guidelines and regulations, and all procedures involving animals were approved by the local Ethics Committee in Animal Experiment and the French Ministry of Higher Education, Research and Innovation (APAFIS#3066-201511301610897 v2).

Our study was performed on 45 primiparous Prim’Holstein cows, in order to limit the influence of previous infectious episodes or antibiotic treatments. None of the animals had health problems or received antibiotic treatment in the year preceding the experiment. Animals arose from two divergent lines with a different susceptibility to mastitis (resistant and control) [21, 22]. These lines were produced by using bulls with contrasted breeding values (scores) for the susceptibility to mastitis (SM). The SM score, as defined by the French national genomic evaluation system is a combination of somatic cell score (SCS) and clinical mastitis (CM) indexes and is based on several thousands of quantitative trait loci. These cows were then genotyped with a customized chip (Illumina EuroG10k) used in the French national evaluation system that estimates genomic breeding values for about 40 traits including susceptibility to mastitis (SM) and body condition (BC) [23]. The list of cows, their genotypes (SM and BC scores), housing conditions and diets are detailed on Additional file 1.

Cows were allotted into three groups based on their expected delivery dates. Sampling was performed at four time points: 1-week pre-partum and 1, 3 and 7 months post-partum (hereafter referred to as -1W, 1 M, 3 M, 7 M). Groups 1, 2 and 3 included respectively 18, 15 and 12 cows. and included both high and low SM and BC scores. Due to differences in calving dates throughout the year, some variations in diets and housing conditions exist between the three groups (see Additional file 1).

Cows were raised together in the same herd under the same management conditions. They were kept indoors (free-stalls) from 1-week pre-partum to at least 3 months post-partum in deep litter housing with daily mulching and fed at 8 am. At the end of the experimental period (7 months), cows were outdoors grazing except for milking. Sampling at 7 M occurred 54, 76 and 110 days after cows started grazing for the groups of animals G1, G2, G3 respectively. Cows were milked twice daily at 06:45 and 15:45. Classical hygienic procedures were used, including cleaning of teats with individual paper towels before milking and post-milking teat dipping in iodine solution.

### Sample collection

One sample per site (Oral, Vaginal and Nasal) and one sample per quarter (Milk) was collected at each timepoint on the 45 cows. Samples from the oral, nasal and vaginal cavities were collected at all sampling times and foremilk samples were collected at 1 M, 3 M and 7 M. At each sampling time, all samples were collected on the same day. The foremilk microbiota [hereafter referred to as milk microbiota (M)] was considered a proxy of the teat cistern and streak canal [4]. The oral microbiota was considered a proxy of the rumen microbiota. Although the oral microbiota does not completely overlap that of the rumen, it was shown to reflect changes in the rumen microbiota [24, 25]. These less invasive sampling procedures are alternatives complying with the refinement principle of the “3R” (replacement, reduction, refinement) rules for animal welfare. Sampling of milk was performed prior to morning milking. Sampling of oral, nasal and vaginal cavities were performed between morning and evening milking (i.e. at 10 a.m. or 2 p.m., always by the same skilled agents (see Additional file 1).

Sampling of milk was performed as previously described [26]. Each quarter was sampled separately. Briefly, teats were thoroughly washed with water and cleaned with 70% ethanol and individual paper towels. Foremilk samples, corresponding to the milk stored in the teat cistern, were collected in sterile plastic tubes, stored on ice for ~ 3 h during transport to the laboratory and stored at − 20 °C until processing for microbiota analysis.

Vaginal sampling was performed at all sampling times except for group 1 at -1W. Cows were restrained, the perineum and vulva were cleaned with water and an iodine solution, and then dried with a paper towel. The lips of the vulva were opened, a disinfected speculum was inserted and a sterile swab (Puritan, Guilford, USA) was used to rub a single site at the bottom of the vagina close to the cervix during 15 s. The swab was withdrawn without touching any vaginal surface and the tip was cut into a sterile tube and immediately stored at − 20 °C until processing.

For oral sampling, the mouth was cleaned with paper towel and a swab (Puritan) was inserted inside the mouth of the cow and rubbed against the left side cheek during 15 s. Then it was carefully withdrawn, the tip of the swab was cut into a sterile tube and immediately stored at − 20 °C until processing. For nasal sampling, the nose was cleaned with a paper towel and the swab (Puritan) was inserted inside the left nostril (approximatively 15 cm), then rubbed against the mucous membrane during 15 s. The tip of the swab was cut and put in a sterile 0.9% NaCl solution during 20 min. Finally, the tube containing the swab in the solution was shaken, the tip removed and the solution stored at − 20 °C.

### DNA extraction and amplicon sequencing

Milk samples (3 mL) were mixed with 1 mL of sodium citrate (1 M, pH 7.5) and centrifuged (18,000*g*, 20 min, 4 °C). The pellet was washed in 1 mL of sodium citrate (20 g/L, pH 7.5), centrifuged (18,000*g*, 15 min, 4 °C) and resuspended in 100 µL TE buffer (10 mM TRIS–HCl (pH8), 2 mM EDTA). Oral and vaginal samples were resuspended by vortexing the swab in 1 mL TE buffer. The swab was removed, the suspension was centrifuged (18,000*g*, 5 min, 4 °C) and the pellet resuspended in 100 µL TE buffer. Nasal samples were centrifuged (18,000*g*, 5 min, 4 °C) and the pellet was resuspended in 100 µL TE buffer.

DNA extraction was performed as described by Knudsen et al. [27] with the following modifications. The bacterial suspension was lysed in 400-μL lysis buffer containing 20 mM TRIS–HCl (pH 8), 2 mM EDTA, 1% Triton X100 and 0.4 g of 0.1 mM zirconium beads (VWR, Fontenay-sous-Bois, France) for 3 × 30 s at 6800 rpm by using a Precellys Evolution device (Bertin Technology, Montigny-le-Bretonneux, France). Following incubation at 95 °C for 7 min, samples were mixed for 15 s and centrifuged (18,000*g*, 5 min, Room Temperature). Proteinase K treatment and DNA purification were performed using the Qiagen kit QIAamp Fast DNA stool mini kit (Qiagen, Courtaboeuf, France), according to the manufacturer’s recommendations. Negative controls undergoing all the extraction steps but without bacterial suspension were included for each set of extractions. This resulted in 66 negative controls that were further amplified and sequenced in order to determine the kitome corresponding to potential contaminant Amplicon sequence variants (ASV) originating from extraction, amplification and sequencing steps.

PCR amplification and sequencing were performed by Genome Quebec (Montreal, Canada). Illumina amplicon library was prepared through a 2-Step PCR amplification. In brief, PCR amplification of the V3-4 region of 16S rRNA gene was done using the universal primers S-D-Bact-0341-b-S-17 and S-D-Bact-0785-a-A-21 [28], in a 10-µL final volume containing 0.45 µM primers, 0.2 mM dNTP, 5% DMSO, 1X Q5 reaction Buffer, 1 µL DNA sample and 0.2U Q5 HiFi polymerase (New England Biolabs, Evry, France). The PCR conditions were as follows: denaturation step at 98 °C for 30 s, followed by 30 cycles of denaturation at 98 °C for 10 s, annealing at 58 °C for 15 s, and extension at 72 °C for 30 s and a final extension at 72 °C for 2 min. Blank controls in which no DNA was added to the reaction were performed. Amplicon quality was checked on 1% agarose gel in 0.5X TBE. No amplicon was visible with blank control. A second PCR was performed to introduce barcodes. After standardization to the same concentration, samples were pooled for sequencing on the Illumina MiSeq PE250 platform (Illumina Inc., San Diego, CA, USA). A total of 1017 samples (excluding controls) were sequenced over the 1080 that could have been sequenced on the 45 cows. Few samples were not collected, including the vaginal samples of the first group of animals 1 week pre partum for practical reasons and some samples did not succeed in the 16 s amplification step and were not sequenced.

### Bioinformatics analysis

Data were analyzed using a combination of the DADA2 pipeline (v. 1.12) [29] and the FROGS pipeline (v3.1.0) [30], following their respective guidelines. DADA2 retrieves biological sequences from reads by modeling the sequencing error distribution. Once the reads were quality checked, the core-denoising algorithm of DADA2 was performed on the forward and reverse reads separately. Amplicon sequence variants (ASV) were then inferred and pairs merged to construct the ASV table. Then, FROGS was used to remove chimera with vsearch [31], using a cross-sample validation strategy: chimera detection was performed independently in each sample and only ASVs detected as chimera in all samples were filtered out. Sequencing depth before and following processing steps is presented on Additional file 2, as well as the rarefaction curves, that were shown to flatten, indicating that sequencing was deep enough to estimate the bacterial composition.

Taxonomic classification was performed with Blastn+ [32], using Silva 132 database [33]. Finally, a phylogenetic tree was inferred from ASV sequences using FastTree 2 [34].

### Statistical analysis

Statistical analyses were performed using R (v 4.0.4) [35] and specialized packages: phyloseq (v. 1.34), DESeq2 (v 1.30.1) [36, 37] and custom scripts [38]. The data were filtered to remove ASV with a low relative abundance (< 1e−5 in each site at each time point or < 5e−5 overall) or a low prevalence (< 5% for each site or < 3 samples overall). To remove kitome ASVs, the samples were split by site, including negative controls as an extra site and the relative abundance of each ASV was computed in each site. ASVs with (1) prevalence higher than 50% in the negative controls and for which (2) the relative abundance in the controls was higher than in all other site combined were flagged as kitome ASVs and filtered out. Finally, all samples with less than 1000 reads after filtering were discarded. Data were rarefied to the same depth before computing alpha and beta diversity indices but not for differential abundance studies.

Alpha-diversity analyses were performed on the observed richness and the Shannon indices. For each index, we compared the diversity across anatomic sites at each time point using a one-way analysis of variance (ANOVA) followed by Tukey's HSD post-hoc test to find significant pairwise differences (adjusted *P* value < 0.05). We also adjusted, for each index, a mixed effect model to the global dataset using sampling time, anatomic site, group of animals (which combines several parameters including housing, diet and season), scores of susceptibility to mastitis (SM score) and body condition (BC score) and the interaction between anatomic site and SM score as fixed effects and animal as a random effect. The R^2^ of each effect was computed using the conditional R2 method implemented in the r.squaredGLMM function from the MuMIn package by comparing the full model to the pruned model.

Beta diversity analyses were performed on the Jaccard, Bray–Curtis, UniFrac and wUniFrac dissimilarities. A Multi-Dimensional Scaling (MDS) was performed on the distance matrix to represent the samples on the principal plane and identify influencing factors. Animal microbiota were clustered independently at each time point and at each anatomic site using hierarchical clustering (ward linkage) on the Bray–Curtis distance matrix. The Adjusted Rand Index (ARI), a measure of similarity between clusterings, was computed to assess whether the clusters reconstructed at different time points were stable and included the same cows. The impacts of several factors on the microbiota was assessed using permutational multivariate analysis of variance (PERMANOVA), as implemented in the adonis2 function from the vegan package. Due to the inclusion of multiple covariates at once, including continuous ones, in the model, a test of homogeneity of dispersion could not be performed for complex models. Significant differences may thus both capture differences in mean composition and differences in beta-dispersion. When a single discrete factor was tested, the PERMANOVA was completed by a permutation test of homogeneity of dispersion, as implemented in the betadisper and permutest functions from the vegan package, followed by Tukey's HSD post-hoc test to find significant pairwise differences.

A differential abundance analysis was performed with DESeq2 [36] at each site to find ASVs whose abundances changed over time. The core microbiota of each anatomic site at each time point was computed as the set of all ASVs with relative abundance higher than 0.01% in at least 50% of the animals. Finally, anatomic sites were compared within each animal at each time point by computing the number of shared ASVs between sites, their proportion of the total number of ASVs in each site and their relative abundance in each site.

### Data availability

DNA sequence datasets are available at the Sequence Read Archive of the National Center for Biotechnology Information under the accession number PRJNA 875059.

## Results

**Fig. 1:**
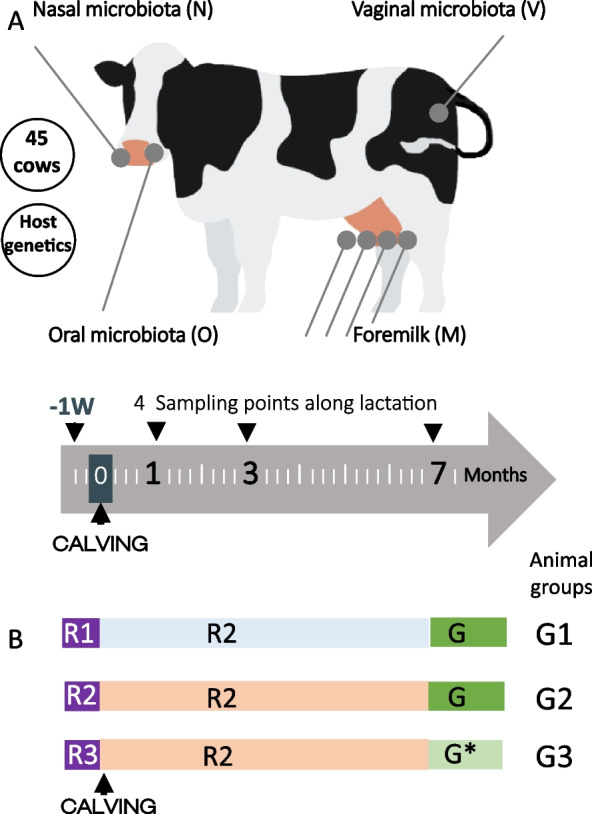
Experimental design and associated metadata. **A** Microbiota associated to four different anatomic sites, namely oral, nasal and vaginal cavities as well as the internal teat cavity/foremilk (hereafter referred as to milk) was characterized on 45 primiparous cows at four different time points, 1 week before parturition (-1W, except for milk) and 1, 3 and 7 months post-partum (1 M, 3 M, 7 M). The relationship between microbiota and host genetics was considered by using cows with different scores of susceptibility to mastitis (SM score). These cows arose from two divergent lines of cows resistant or susceptible to mastitis [21]. An additional score was included, corresponding to the body condition (BC). **B** Diet (rations R1, R2, R3 or G—grazing) and housing conditions (purple: indoor housing 1, blue: indoor housing 2, orange: indoor housing 3, green: grazing) for the 3 groups of animals (see Additional file 1 for details on housing and diet). *Grazing combined with corn silage

The animal experiment was designed to address the composition, dynamics and the presence of common microbes in the microbiotas associated to the oral, nasal, vaginal and internal teat cavities of dairy cows. A main objective was to explore and compare their variability, within and among animals, over a period of 7 months, allowing to cover two major physiological periods that are calving and lactation (Fig. 1). Additionally, we used Prim’Holstein cows with different genetic susceptibility to mastitis [21] that allowed us to highlight the role of host genetics on microbiota composition. Overall, a total of 1017 samples from 45 cows were collected and subjected to metataxonomics using the 16S rRNA gene (see “Material and methods” section). Metadata associated to this dataset include the animal ID, genetic scores for mastitis susceptibility and body condition, as well as changes in environmental conditions including housing and diet. Likewise, the “group of animals” factor combines several additional parameters including housing, diet, sampling time or season (see Additional file 1).

Overall, our sequencing effort produced a total of ~ 30 millions read pairs. Data were further filtered to remove (1) ASVs with no bacterial or archaeal affiliation at the Kingdom level (e.g. contamination from *Bos taurus*), (2) ASVs with low abundance, low prevalence or belonging to the kitome and (3) samples with an insufficient number of reads (< 1000) (see material and methods section for a complete description of the filters used). This resulted in 12,928,908 quality filtered sequences corresponding to a total of 921 samples: 158 oral (O), 168 nasal (N), 134 vaginal (V) and 461 milk (M) samples. This corresponded to an average of 14,038 sequences per sample, and a total of 1209 ASVs detected based on a minimum abundance of > 1 × 10^−5^. Overall, the sequencing coverage ranged between 1003 and 81,591 reads per sample, with a mean depth being slightly higher in nasal compared to oral and vaginal, and to milk samples (see Additional file 2A). Out of the 545 ASVs detected in control samples, 433 were identified as kitome ASVs (Additional file 13). For each of the remaining 112 ASVs, the mean abundance in the negative controls was less than 1%, except for two with mean abundances between 1 and 2%. By contrast, those ASVs were found at higher proportions in at least one of the body sites, suggesting they are non-contaminant ASVs and were thus not removed. Kitome removal had a low to moderate impact on body sites samples: the median fraction or reads filtered out was less than 1.8% for oral and nasal samples, 5.2% for vaginal samples and 15.3% in milk samples, as expected from their lower bacterial load. By contrast, it filtered out 89% of the reads in the control samples (see Additional file 2B). Sequencing was deep enough to estimate the bacterial composition, as revealed by the rarefaction curves that were shown to flatten (see Additional file 2C).

### Characteristics of milk, oral, nasal and vaginal microbial communities

**Fig. 2:**
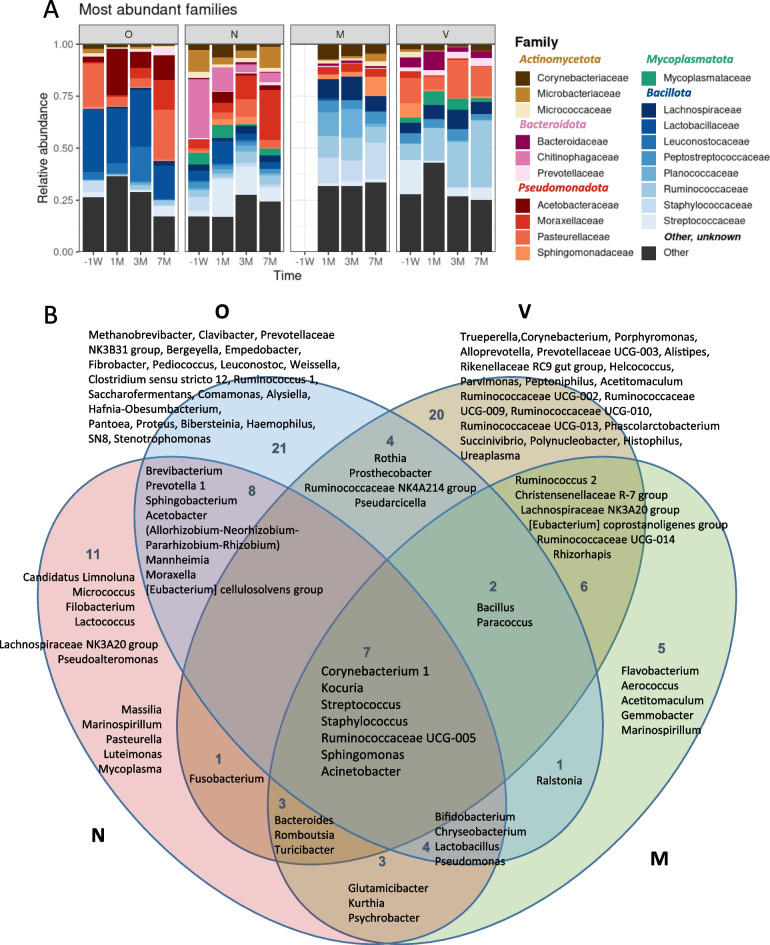
Overview of the cow nasal (N), oral (O) vaginal (V) and milk (M) microbiota. **A** Mean bovine taxonomic profiles associated to the cow nasal, oral and vaginal cavities and milk 1 week before calving (-1W) and 1, 3 and 7 months (1 M, 3 M, 7 M) post-partum. The 20 dominant families are presented. Families belonging to Actinomycetota (formerly named Actinobacteria) are displayed in shades of brown, Pseudomonadota (formerly named Proteobacteria) in shades of red, Bacteroidota (formerly named Bacteroidetes) in shades of pink, Mycoplasmatota (formerly named Tenericutes) in shades of green and Bacillota (formely named Firmicutes) in shades of blue. **B** Venn diagram combining the 25 dominant genera for each anatomic site at each time point (based on Additional file 3)

The microbes identified were affiliated to 20 phyla but five phyla dominated on all four sites. These were the Bacillota (formerly named Firmicutes), Pseudomonadota (formerly named Proteobacteria), Bacteroidota (formerly named Bacteroidetes), Actinomycetota (formerly named Actinobacteria) and Mycoplasmatota (formerly named Tenericutes) phyla, with the dominant families being presented on Fig. 2A. Although most of these families were shared between the four anatomical sites, their mean relative abundance was site and time dependent (Fig. 2A). More specifically, the oral microbiota was dominated by the Lactobacillaceae and Leuconostocaceae of the Bacillota and by the Acetobacteraceae, Moraxellaceae, and Pasteurellaceae of the Pseudomonadota. The nasal and the vaginal microbiotas displayed more complex profiles, both being characterized by the presence of members of the Mycoplasmataceae family that are absent in the other microbiotas. Yet, the nasal microbiota was marked by a relatively high abundance of Chitinophagaceae of the Bacteroidota while the vaginal microbiota had a relative high abundance of Bacteroidaceae when compared to the three other sites. Finally, the milk microbiota was dominated by families belonging to Bacillota, including Lachnospiraceae, Peptostreptococcaceae, Planococcaceae, Ruminococcaceae and Staphylococcaceae.

For each anatomical site and time point, the 25 most abundant genera were identified (see Additional file 3). When dominant, these genera were also highly prevalent (median prevalence of 42%), but only seven genera were found concomitantly in all sites (Fig. 2B). These were the *Corynebacterium, Kocuria, Streptococcus, Staphylococcus*, Ruminococcaceae UCG-005, *Sphingomonas* and *Acinetobacter* genera. Several others were found across two or three sites, with slightly more of the dominant bacterial genera being common to the oral and nasal microbiotas, or to the milk and vaginal microbiotas. Finally, several dominant genera were associated to only one anatomical site, with the highest number displayed by the oral and vaginal microbiotas.

### The bovine microbiota changed with site and time, whereas it was poorly related to genotype

**Fig. 3:**
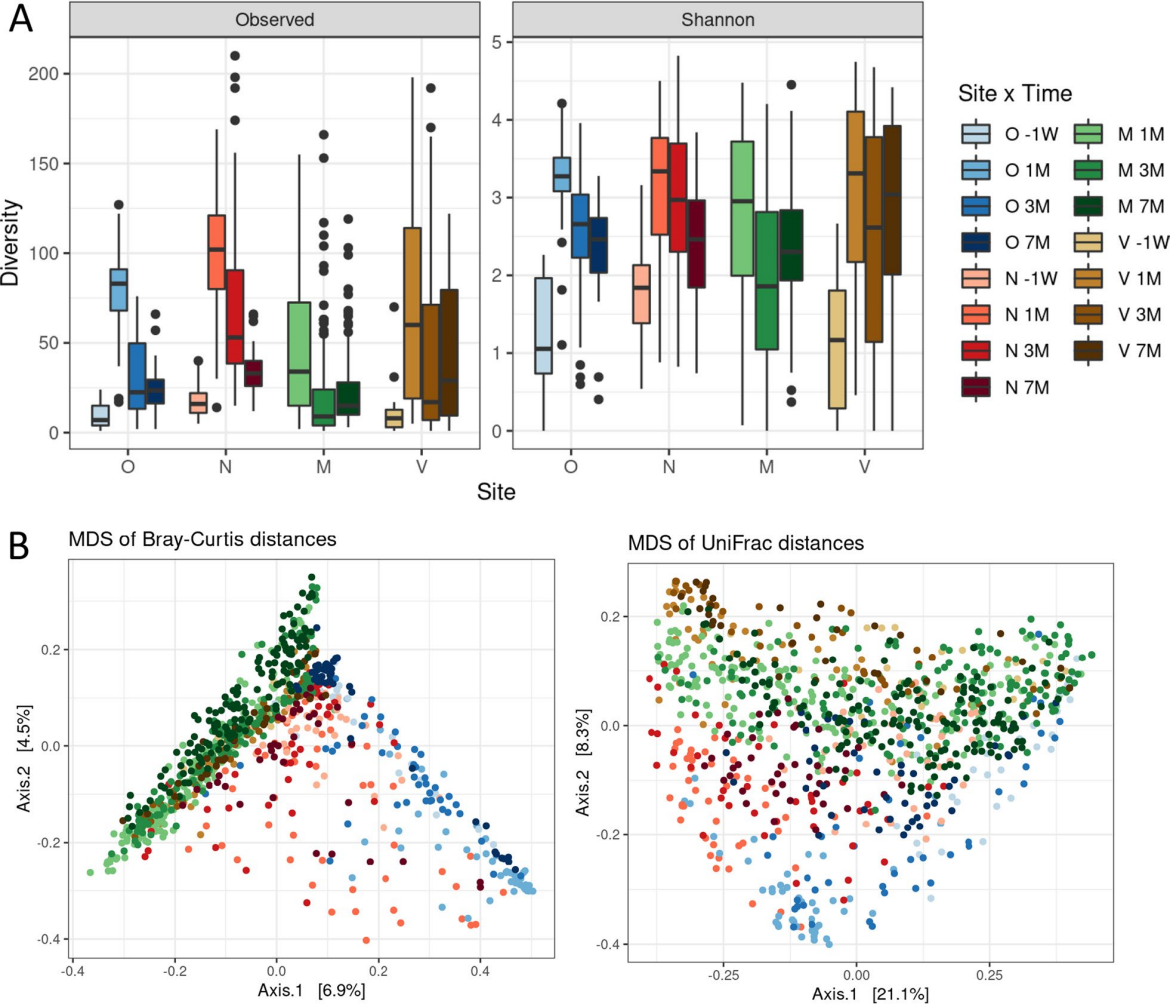
The sites differ in terms of richness and repertoire.** A** Alpha-diversity of the cow nasal (N), oral (O), vaginal (V) and milk (M) microbiota 1 week before calving (-1W) and 1, 3 and 7 months (1 M, 3 M, 7 M) post-partum. The distribution of two diversity indices, namely the observed richness and the Shannon index, indicates both a site and a time effect on the alpha-diversity. **B** Multi-Dimensional Scaling (MDS) of bovine microbiota associated to the four anatomic sites and at four different time points during lactation. MDS was performed based on the measurement of the Bray–Curtis or UniFrac distances. Samples are indicated by points and colored with regard to the anatomic sites (O, N, V, M) and the four time points (-1W, 1 M, 3 M, 7 M) (see legend panel A). PERMANOVA performed with both distances revealed a significant site effect on beta-diversity, although the p-values can be anti-conservatives due to differences in beta-dispersion across time (betadisper *P* val < 0.01, see Additional file 11). Color codes (displayed right side in panel **A**) are similar in panel **A** and **B**

Multivariate adonis models were built to assess the influence of the anatomic site and time, as well as susceptibility to mastitis (SM) and body condition (BC) scores, individual animal (ID) and group of animals on the beta-diversity distance matrices (Table 1). The cow microbiota was strongly related to the anatomical site, with a contribution of this factor to the beta diversity of microbiotas between 4.8 and 13.2% depending on the distance matrix used. The beta-diversity analysis using MDS showed a separation between oral samples, nasal samples and milk/vaginal samples that grouped together (Fig. 3B). The “site” effect was found as significant by PERMANOVA at each time point using all distances (*P* val < 1e−4). The *P* values are anti-conservative due to differences in beta-dispersion (betadisper *P* val < 0.007, see Additional file 11) but the site effect is highly visible (see Additional file 12). The "site" factor contribution was evaluated to be 10.4%, 16.4%, 9.3% and 11.5% at -1W, 1 M, 3 M and 7 M respectively with Bray–Curtis distance, and 18.1%, 19.2%, 15.4% and 17.3% at -1W, 1 M, 3 M and 7 M respectively with UniFrac distance. Also, a significant effect was obtained when considering the “time” that contributed to 2.4–6.6% to the variation. The cow’s microbiota was also significantly related to the “group of animals” factor but it only explained ~ 0.5% of the variation. Interestingly, a significant “animal ID” effect was revealed with the Bray–Curtis distance (and a trend with wUniFrac), with a contribution around 4%. In contrast, the microbiota was poorly related to the SM and BC scores. A significant effect was observed only for the SM score with the Unifrac distance, together with a trend for the Jaccard distance, but its contribution was less than 0.2%. Interestingly, analyses performed by site revealed a significant impact of the SM score on the beta-diversity of milk microbiota only (*P* val ≤ 0.02 with the Unifrac and Jaccard distances, with a contribution of 0.3–0.4%; data not shown), while no significant effect of the SM score was observed on the other sites (*P* val = 0.22–0.96). Similar results were obtained when converting the continuous SM score factor in a discrete one, considering 3 groups of animals (SM score < − 1, SM score > 1 and the others): SM score impact was observed on milk microbiota only (*P* val < 0.024 with the Unifrac, Jaccard and Bray Curtis distances, with a contribution of 0.5–0.8%) but this impact could result from lower beta dispersion (betadisper *P* val = 0.04) in the high SM score group compared to the two others.

**Table 1:** Effects of different factors on the beta diversity of dairy cow microbiota

	Jaccard^a^		Bray–Curtis^a^		UniFrac^a^		wUniFrac^a^	
	R2^b^	*P* value	R2^b^	*P* value	R2^b^	*P* value	R2^b^	*P* value
Group of animals	0.0047^b^	0.001***	0.0053	0.001***	0.0046	0.001***	0.0045	0.002**
Site	0.0484	0.001***	0.0768	0.001***	0.1119	0.001***	0.1324	0.001***
Time	0.0242	0.001***	0.0266	0.001***	0.0663	0.001***	0.0375	0.001***
SM_score	0.0012	0.061.	0.0010	0.423	0.0017	0.035*	0.0006	0.786
BC_score	0.0011	0.206	0.0012	0.122	0.0009	0.433	0.0007	0.716
Animal	0.0411	0.166	0.0449	0.001	0.0381	0.129	0.0400	0.054.

^a^Summary of the effects (*P* values) (as determined using Jaccard, Bray–Curtis, UniFrac and wUnifrac distances) of different factors on the beta diversity of the oral, nasal, vaginal and milk microbiota. ***P value < 0.001; **P value < 0.01; *P value < 0.05; “.” *P* value < 0.1

^b^R2 indicates the contribution of each factor to the beta-diversity. A R2 of 0.0047 indicates a contribution of the “group of animals” factor to the beta-diversity of 0.47% with the Jaccard distance

The analysis of the relationships between the different factors and cow microbiota was completed by the effect of these factors on alpha-diversity (Table 2). The observed richness of cow microbiota differs between sites, being higher for nasal, intermediate for oral and vaginal, and lower for milk microbiota (Fig. 3A, Table 2). It also changes with time and with the SM score. A significant effect on the Shannon index was only reported for time (and a trend for SM score). Impact of SM score on alpha-diversity was low (1% and 0.5% for observed richness and Shannon indices respectively) and not modulated by the anatomic site (no interaction between both factors).

**Table 2:** Effects of different factors on the alpha diversity of dairy cow microbiota

Factor	Observed richness^a^	Shannon^a^
Group of animals	0.9104	0.4935
Site	< 0.0001***^b^	0.1438
Time	< 0.0001***	< 0.0001***
SM_score	0.0039**	0.0576.
BC_score	0.0748.	0.4776
Site:SM_score	0.4616	0.7353

^a^Summary of the effects (*P* values) of different factors on the alpha diversity (observed richness and Shannon index) of the oral, nasal, vaginal and milk microbiota. ***P value < 0.001; **P value < 0.01; *P value < 0.05; “.” *P* value < 0.1

^b^Site effect on observed richness depends on time point: at -1W: Nasal > Oral; at 1 M: Nasal-Oral > Vaginal > Milk; at 3 M: Nasal > Oral-Vaginal > Milk; at 7 M: Nasal-Vaginal > Oral-Milk

**Fig. 4:**
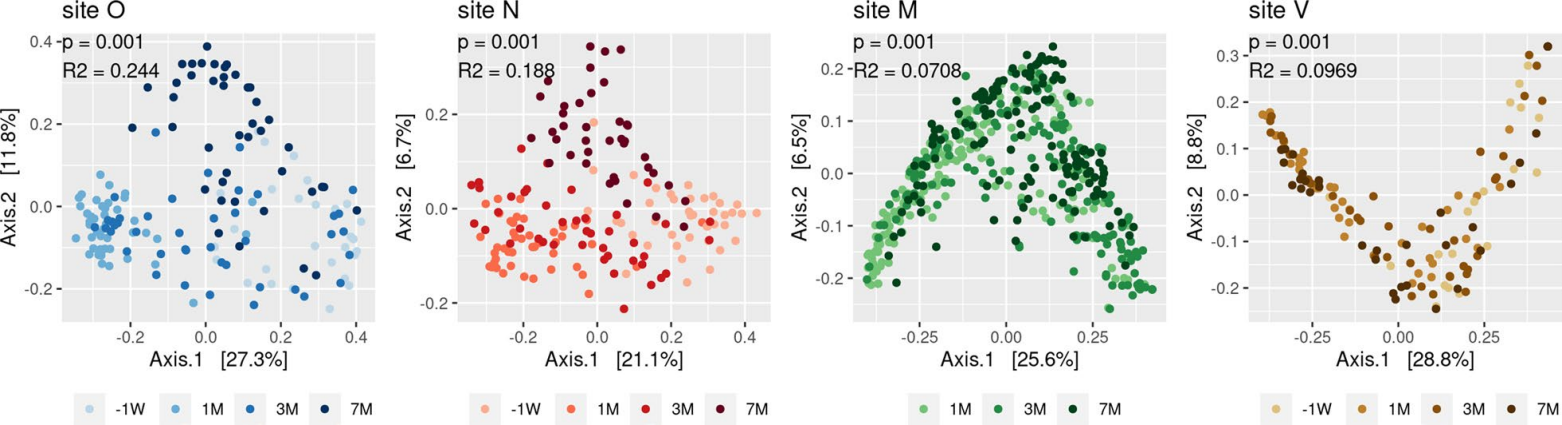
Temporal variation of bovine oral, nasal, vaginal and milk microbiota during lactation is site dependent. For each site, multi-Dimensional Scaling (MDS) was performed based on the measurement of the UniFrac distance. Samples are indicated by points and colored with regard to the four time points (-1W, 1 M, 3 M, 7 M). Permanova performed with the UniFrac distances revealed a time effect on the microbial composition associated to each site with a higher contribution of time to oral and nasal microbiota beta-diversity compared to milk and vaginal. (see *P* value and R2 indicated on the figure; A R2 of 0.244 (first panel) indicates a contribution of time to oral microbiota beta-diversity of 24.4%, although that number may be inflated by differences in beta dispersion). Similar results were obtained with Jaccard, Unifrac and wUniFrac distances

Considering changes in microbiota composition with time, we further explored these changes for each site (Fig. 4). Microbiota composition changed with time in all sites, albeit at different degrees. Larger variations between time-series were observed in oral (24%) and nasal (19%) microbiota beta-diversity whereas it was moderate in vaginal and milk (10% and 7% respectively, Fig. 4). The most important changes occurred between 1-week pre-partum and 1 month post-partum where up to 90% of differentially abundant ASVs were detected, depending on the site. Changes during the lactation period were less important in all sites including milk. Additional file 4 shows differentially abundant ASVs per time and site.

Regarding the relationship between milk microbiota and SM score, a differential analysis between low, medium and high SM scores was done, returning 29 ASVs differentially abundant in the milk microbiota with respect to the SM score category (SM score < − 1, SM score > 1 and the others) (see Additional file 4). The differences were however not systematic and these ASVs cannot be used as biomarkers as the median count of all those ASVs was 0 (prevalence < 30%) for each SM score category.

We then determined the clustering of microbiotas for each site and time point for assessing the temporal stability of microbiotas at each site. Positive correlation between the different time points was observed for the oral microbiota, and to a much lesser extent for the nasal and milk microbiotas, while no positive correlation was observed for vaginal microbiota (Additional file 5). This suggests a similar evolution during lactation of oral microbiotas that were initially close (i.e. that belonged to the same cluster). This co-clustering observed in the oral microbiota was likely due to a “group of animals” effect, and thus to the environmental conditions of each group of animals, as highlighted by connecting lines that represented groups of animals.

### A limited number of core ASVs between cows contrast with the presence of shared ASVs between anatomical sites in individual cows

We compared the core microbiota associated to each site at each time point for assessing the similarity of the microbiota within the herd. The core microbiota was defined as the set of ASVs having a relative abundance higher than 0.01% in more than 50% of the animals. Only a few core ASVs were obtained for each site with a maximum observed at 1 M post-partum, mainly in the oral and nasal microbiota (see Additional files 6 and 7). At 1 M post-partum, the nasal, oral, vaginal and milk core microbiota included 48, 40, 6 and 3 core ASVs, respectively, representing from 0.3% in milk up to 7% in oral cavity of the total number of ASVs that were present in each site at this time point. Further comparison between the core microbiota associated to the different sites indicates that the core microbiota of each site was specific with only few core-ASVs shared between two or three sites, mainly between oral and nasal microbiota, and none was found across the four sites. In contrast, at the individual cow level, the proportion of these core ASVs was greater, reaching at 1 month 28.5% and 32% in the nasal and oral microbiota, respectively, whereas it was 7.1% and 5.4% in the vaginal and milk microbiota, respectively (see Additional file 6).

Several of the core ASVs corresponded to highly abundant ASVs. Thirty-two out of the 78 core ASVs at 1 M, 18 out of 24 core ASVs at 3 M and 9 out of 14 ASVs at 7 M belonged to the 50 most abundant ASVs. Several core ASVs identified at 3 M (22 out of 24) or 7 M (7 out of 14) were also identified as core ASVs at 1 M, suggesting a persistency of these core ASV from 1 to 7 months.

**Fig. 5:**
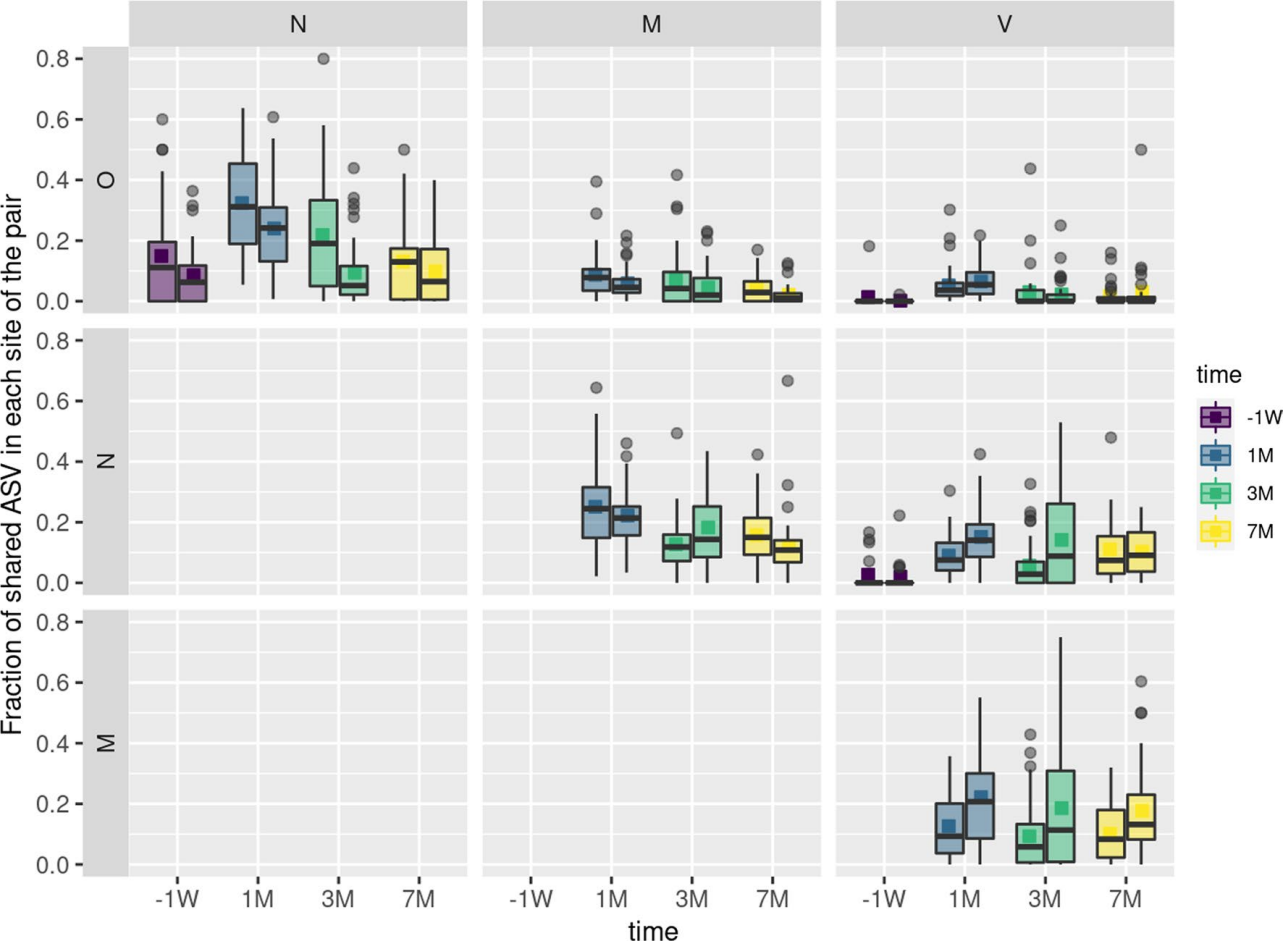
Fraction of shared ASVs between the different anatomic sites at the animal level. For each animal and each time point, the number of ASVs shared between two sites was calculated and divided by the total number of ASVs in each site of the pair. Distribution of these fractions of shared ASVs between two sites is presented as boxplot. For each pair of sites and each time point, the left boxplot corresponds to the fraction of shared ASVs in the site mentioned in line and the right boxplot corresponds to the fraction of shared ASVs in the site mentioned in column. Boxplot median and colored square represent the median and mean fractions of ASVs shared between the two sites respectively

Moving from the herd to the animal level, we analyzed ASV sharing between the different sites within each individual cow. A large proportion of ASVs was shared between the oral and nasal microbiotas of each cow. Up to 32% of the oral microbiota, representing up to 57% of the relative abundance, was common with the nasal microbiota. Conversely, up to 24% of the nasal community, representing up to 29% of the relative abundance, was common with the oral microbiota (Fig. 5 and Additional file 8). There was also important sharing between the nasal and milk microbiota (up to 25% of the ASVs, up to 34% of the relative abundance) and between the milk and vaginal microbiota (up to 22% of the ASVs, up to 25% of the relative abundance). In contrast, nasal and vaginal microbiota shared few ASVs before parturition (2–3%) but the proportion increased to up to 15% during lactation. Shared ASVs between oral and milk or vaginal microbiotas were less frequent with a maximum of 9% and 6% common ASVs for milk and vaginal microbiotas, respectively.

There were 6927 occurrences of shared ASVs between two sites of the same cow. The number of shared ASVs between three or four sites, although several folds lower, was still considerable. There were 576 occurrences in which an ASV was shared by 3 sites of the same cow at a given time point, and 59 occurrences in which it was shared between all four sites (see Additional file 9). Most ASVs shared by three or more sites of a same animal were found in nasal, vaginal and milk microbiota (~ 50%), and to a lesser extent in oral, nasal and milk microbiota (~ 24%). The occurrence of common microbes between two or more microbiotas was observed for nearby but also distant sites. ASVs shared by three or more sites corresponded to 151 distinct ASVs. Most of them (98 out of the 151 distinct ASVs) were specific of one or two animals, whereas a limited subset of 13 ASVs were shared by three or more sites in more than ten animals (from eleven to forty-one animals) (see Additional file 10). These 13 ASVs were among the 50 dominant ASVs of the dataset.

## Discussion

In this study, we investigated the microbiota associated to four different anatomic sites throughout the lactation of primiparous dairy cows with a different predicted susceptibility to mastitis. To our knowledge, this is the first longitudinal study that explores simultaneously the microbiota of dairy cows associated to various anatomical sites.

This longitudinal and multisite characterization of dairy cow microbiota points out a major effect of site and time on cow microbiota composition and richness as well as an animal ID effect. An original aspect of the study was that cows had contrasted genotypes for susceptibility to mastitis and body condition (SM and BC scores). Cow microbiotas were not associated with these scores, except the milk microbiota which was significantly related to the SM score (*P* val < 0.05), but with a very low contribution of this factor to the beta-diversity (0.3–0.8%), compared to the site and time factors.

As expected from the literature [13–16, 39–42], microbiota differed between anatomic site in terms of richness (alpha-diversity) and composition (beta-diversity). The greater similarity observed between vaginal and milk microbiotas than between other body sites (Fig. 3B) was also reported in goats [43]. Such site-specific microbial composition was even observed for closely related anatomic sites of the respiratory tracts [12], the mammary gland [44], or the digestive tract in adults or calves [11, 15]. This specificity of microbiota is due to the nutritional and physico-chemical conditions associated to these sites such as pH and oxygen concentrations but also to differences in terms of tissue composition and local immune system. Also, exposure of these sites to the external environment and diet likely has a modulating effect on these microbiotas [5, 45–47]. For instance, the high abundance of lactic acid bacteria in the oral microbiota found in this study probably originated from the silage in the diet.

Besides, dairy cow microbiota underwent several changes over this 7 month-period, likely in response to internal (physiological changes) or external (environmental changes) stimuli. Microbiota plasticity was notably observed during rumen and nasopharyngeal microbiota assembly in calves and changes in these microbiota composition were associated to nutritional transitions [15, 48] and to arrival to a new feedlot [18, 49]. Microbiotas from the vaginal, uterus and feces were previously found to vary in relation to the estrous cycle in heifers and to fertility [17, 50, 51]. In the present study, important modifications of the bovine microbiota were observed around the critical period of calving and during lactation. Interestingly, several changes in ASVs abundance occurred between 1 week before calving and 1 month of lactation, which might be related to physiological changes during the transition period, especially for reproductive organs. Drastic changes were reported in the vaginal and uterine microbiotas around parturition [2, 52]. Milk microbiota composition changes during lactation as well, notably during the first weeks of lactation [8, 53]. Beside physiological changes, modifications in housing conditions and diet may contribute to the so-called “time effect” on microbiota. Doyle et al. [45] also observed an effect of the seasonal housing, corresponding to indoor and outdoor environments on raw milk, teat skin and feces microbiota. Likewise, changes in milk microbiota have been associated to housing conditions, including the bedding type [54] and the grazing systems [46]. Among environmental factors, diet was found to influence nasal [47], rumen [5, 48, 55, 56] and milk microbiota [57, 58]. In the present study, the influence of the diet was notably illustrated through the decrease of several lactic acid bacteria ASVs abundance in oral and nasal microbiotas at 7 months, resulting from diet shift from silage to grazing. The oral and nasal microbiotas were more variable than those from the vagina and milk, which might be due, as mentioned above, to a more direct external exposure to the surrounding environment and diet. This suggests that environment and diet are among the main drivers of microbiota changes observed during the lactation and that farm management practices are potential levers to shape dairy cow microbiota.

### Microbe sharing between sites of the individual animals

The simultaneous exploration of multiple microbiotas allowed to identify microbes that are collectively shared between different anatomic sites of dairy cows. This suggests possible microbial fluxes between these sites or host-driven shaping of the microbiota. Interestingly, our data clearly point out that these shared microbes occur between sites of the individual animals (Fig. 5, and Additional files 8 and 9). We cannot prove, at this point, that ASVs shared between two or more sites of each animal corresponded to common strains. Notwithstanding, ASVs, which correspond to unique sequences of the 16 s rRNA gene V3-V4 amplicon, are more discriminant than the commonly used Operational Taxonomic Units (OTU), which generally consider aggregation of amplicons within a 3% nucleotidic variation. The share of microbes was pronounced between nearby sites such as the nasal and oral cavities, the anatomical proximity favoring microbial fluxes as previously reported for different sites of the respiratory tracts [12]. Yet, microbes were also shared between more distant microbiotas such as vaginal or nasal with milk microbiota. This raises the question of possible fluxes between these sites and the routes that are used. Some of these shared ASVs originate from the environment or the diet and may have reached these different anatomic sites through independent ways. This is likely the case for some of the highly abundant ASVs that were identified in more than three anatomic sites of more than ten animals (see Additional file 10). Some behavioral traits of cows, such as contacts between noses and suckling between cows, as well as microbial transfer via vaginal secretions, feces, rumen regurgitation during rumination may account for external transmission between distant anatomic sites [15]. It is probable that ASVs belonging to taxa commonly associated to the rumen, such as the Ruminococcaceae family, were transferred from the rumen to the others sites. Other genera, such as *Staphylococcus, Streptococcus* and *Corynebacterium*, are more ubiquitous and commonly found in the microbiota of the different sites, suggesting that microbial fluxes between two sites may occur in both directions. Internal routes have also been proposed to explain transmission between distant sites, such as the enteromammary pathway, allowing microorganism delivery from the digestive tract to milk through circulating immune cells [59, 60]. This pathway, which has been mainly investigated in women, but also suggested in cows, would allow a direct microbial transmission from the mother to the offspring through milking, thus contributing to the primo colonization of the offspring gut [15, 42]. Nevertheless, the existence of this route is still a matter of debate as, following translocation into the mammary gland, bacteria would have to face the mammary gland immune system [61]. Besides, most of these studies are based on metagenomics, which does not allow conclusions on the physiological state (alive or dead) of these potentially translocated bacteria. Additional experiments are required to confirm that shared ASVs between different anatomic sites of an animal correspond to common strains and to elucidate the routes of transmission between these sites. Furthermore, it should be noted that ASVs shared between sites within an animal may then have different relative abundances in each site, in relation to the specific environmental conditions encountered.

### Towards the concept of holobiont: animal-specificity of the cow microbiota and relation with host genetics

Our results show that an important proportion of microbes are shared between different anatomic sites of the same cow. One can then wonder whether such sharing exists between different animals from the same herd, in the same environment and fed the same diet. In this study, cows exhibited similar taxonomic profiles for a given site when looking at taxonomic levels such as families or genera, suggesting that they share a large set of microbes. However, analyses performed at the ASV level revealed only a limited number of ASVs widely shared between these animals. This is illustrated by a low proportion of core ASVs for each anatomic site at each time point compared to the diversity of ASVs found in the same site and time point within the herd (< 7%; see Additional file 6). Of note, the core ASVs, defined here using both occurrence (> 50% animals) and abundance (> 0.01%) criteria, did not rely on too stringent/restrictive cutoffs compared to other studies [62]. Several of these core ASVs corresponded to highly abundant ASVs, thus were more easily detected in several animals. Despite their limited number, these abundant core ASVs constitute a significant fraction of the community at a given site and contribute to the site-specificity observed using Bray–Curtis distances. A higher number of core ASVs was observed in oral and nasal compared to vaginal and milk microbiota, probably due to a higher external exposure of the two first anatomic sites to the surrounding environment. Interestingly, several oral and nasal core ASVs at 1 M and 3 M corresponded to lactic acid bacteria that can be naturally present or added as inoculant in the silage, as well as other genera such as *Acetobacter* or spore-forming bacteria which are classical contaminants of silage [63, 64]. These probably silage associated core ASVs were absent at 7 M, when cows were grazing. It would have been of interest to include silage and environmental samples to confirm the origin of several shared ASVs. Cows were also fed with silage 1 week before calving, but the diet differed between the three groups of animals at this time point, which may explain a low number of core ASV in oral and nasal microbiota at -1W. Apart from these diet-associated ASVs, most ASVs shared between animals belong to the nasal microbiota, probably due to frequent contacts between animal noses [65]. These nasal core ASVs mainly belong to the Bacillota including the Clostridia class and *Staphylococcus*, as well as to *Corynebacterium* and *Bifidobacterium*. Most of them were also present in the three other sites, yet at a lower prevalence. Interestingly, Amat et al. recently reported the existence of a limited number of core ASVs (41 ASV) common to a high portion (60%) of rumen, vaginal and nasopharyngeal samples in heifers, corresponding to the same taxa [16].

One of the main result of this study is that, apart from few dominant ASVs, mostly originating from their common environment and diet, animals did not share a large set of ASVs but rather hosted a specific set of microbes, suggesting a tight interplay between each animal and its microbiota [66]. It should be noted that differences in ASVs composition do not mean differences in terms of functionalities. Although microbes differ between animals, they may perform similar functions (metabolism, interaction with the host), as already highlighted in the rumen [67] or in the human microbiome [68, 69]. The animal effect may be related, in part, to the transmission of microbiota from the dam to the calf during calving and in the first stages of life. Dam colostrum, vagina, feces and oral microbiotas have been suggested to contribute to the calf gastrointestinal tract colonization during the first days [13, 15, 39, 43], although strain transmission still needs to be clearly demonstrated. This primo-colonization likely contributes to the shaping of the calf microbiota through direct colonization, competition with microorganisms arising from the surrounding environment and diet (colonization resistance) or by influencing early immune system development and inducing tolerance to a specific set of commensal microbes later in life [39].

Another factor that could account for this host specificity of microbiota composition is genetics. Several studies established a relationship between host genetics and rumen microbiota, with specific gene polymorphisms associated to changes in rumen microbiota composition and further consequences on feed efficiency or methane emission [10, 70]. Likewise, Fan et al. reported a relationship between early gut microbiota and genetics in relation to immunity and metabolism for calves with varying breed composition, raised in the same environment with identical diets [9, 71]. The influence of breed was also observed on milk microbiota using different breeds farmed under the same conditions [53, 72]. Similarly, Derakhshani et al. [8] revealed an association of the bovine leukocyte antigens (BoLA) DRB3.2 gene polymorphism with colostrum microbiota composition of dairy cows. Polymorphism in BoLA has been previously associated with differences in somatic cell counts and mastitis susceptibility [73, 74]. In the present study, cows with different susceptibility to mastitis were used [21]. The SM score, as defined by the French national genomic evaluation system, is based on several thousands of quantitative trait loci and several additional markers spread throughout the genome, including some genes of the BoLA. Impact of the genetic selection was experimentally confirmed on the somatic cell counts and the clinical mastitis rate [21, 22]. Here, a limited but significant relationship was observed between SM score and milk microbiota, while the microbiota associated to the other sites was not influenced by this factor, suggesting a site-specific influence of host genetics on microbiota. Of note, the SM score takes into account polymorphism in some BoLA related genes, but it does not include the BoLA-DRB3 marker used by Derakhshani et al. Instead, the SM score includes other BoLA related genes in the vicinity of BoLA-DRB3 in addition to several additional markers, which precludes a direct comparison with their results. Besides, Derakhshani reported a relationship between the BoLA gene polymorphism and colostrum microbiota only at day zero, while the effect was not significant on milk microbiota collected on subsequent days [8]. In our study, milk was collected from 1 month post-partum onwards, which could also explain the poor effect observed in our case. Whether the animal effect observed on microbiota in this study can be associated to genetics thus requires further investigations.

## Conclusions

This longitudinal study points out changes with time (from calving to 7 months lactation) of the specific microbiota associated to the mouth and nose, and to a lesser extent, vagina and milk of dairy cows. These changes likely resulted from subsequent physiological and environmental (diet, housing) changes. The impact of host genetics on cow microbiota was also suggested, through a low but significant relationship between the SM score and milk microbiota only. Importantly also, a significant share of microbes was observed within an individual host between microbiotas associated to different anatomic sites, including distant ones. In contrast, the microbes shared between cows was limited. Although animals displayed similar taxonomic profiles for a given site, they hosted a specific set of microbes (ASV), suggesting a host control. Several questions still need to be addressed, including the routes, either internal or external, used by shared microorganisms to move between sites and the role of host determinants, including genetics, in shaping the microbiota. This information is needed to devise strategies aiming to modulate the host-microbiota interplay for health and production.

## Supplementary Information

**Additional file 1**. Animal description and associated metadata.

**Additional file 2**. **Sequencing depth. A.** Average sequencing depth in each anatomic site and each time point, before processingand following pre-processing and filtration steps. Dotted lines indicate an average read count of 1000 and 10,000 reads. **B**. Impact of the kitome ASVs removal on the number of reads. Dotted lines indicate an average read count of 1000 and 10,000 reads. **C.** rarefaction curves on processed data.

**Additional file 3**. **Dominant genera in oral, nasal, vaginal and milk microbiota**. For each anatomic site, the 25 more abundant genera at each sampling time are listed with their rank, mean abundance and prevalence at each sampling time. In Bold: mean abundances > 5%, in blue: 25 dominant genera for each site at each time point, and corresponding prevalence.

**Additional file 4**. **List of ASVs with abundances changing over time in oral, nasal, vaginal and milk microbiota, and list of ASVs with abundance changing with SM score category in milk microbiota** as determined by a DESeq2 analysis. Effects are reported as log2 fold changeand adjusted *P* values.

**Additional file 5**. **Alluvial plot of cows across microbial clusters.** The alluvial plot shows how each cowflows across clusters. Ribbons departing from the same cluster and splitting towards several clusters means that the clusters are not conserved across times. Clustering of the microbiota at each site and time point was performed using hierarchical clustering and cutting the dendrogram to have 5 clusters. This number of clusters was chosen so that cluster size allowed pointing out differences in cluster composition. For each site, the distributions of cows into clusters were compared between consecutive time points using the ARI to assess the temporal stability of the clusters. Positive yet very low ARI values, indicative of limited stability, were observed for the nasal microbiota between 1 week before parturition and 1 M and 3 M, and for the milk microbiota between all-time points, while no stability was observed for vaginal microbiota. ARI values were higher for the oral microbiota at all time points, with the highest valueobserved between 1 and 3 M as well as between 3 and 7 M. This stability of the clusters across time points was likely due to a “group of animals” effect, and thus to the environmental conditions of each group of animals, as highlighted by ribbons with the same colormoving together across clusters. Table indicates the Adjusted Rand Indexof animal clustering across time. 1 corresponds to equal clustering whereas 0 is the score expected for two random clusterings.

**Additional file 6**. **Dairy cow core microbiota A.** Venn diagram representing the core ASVs of the cow nasal, oral, vaginaland milkmicrobiota, 1 week before calvingand 1, 3 and 7 monthspost-partum. Core ASVs were defined for each anatomic site and each time point as ASVs whose relative abundance was higher than 0.01% in at least 50% of samples. A very limited number of core ASV was obtained for each site, in particular vaginal and milk microbiota, with no core ASVs shared between the four anatomic sites. **B.** For each site and time point, proportion of the core ASVs compared to the total ASV number found in the site at this time point over the 45 cows, and proportion of the core ASVs compared to the number of ASV found in each cow.

**Additional file 7**. **List of core ASVs of the oral, nasal, vaginal and milk microbiota**. Core ASVs of the 4 anatomic sites are listed for the different time points. Core ASV are defined as ASVs with relative abundance higher than 0.01% in more than 50% of the animals. ASVs in bold correspond to those that belong to the core ASV of a given site at least at 2 time points.

**Additional file 8**. **Relative abundance of shared ASVs between the different anatomic sites at the animal level.** For each animal and each time point, the total relative abundance of shared ASVs between the two sites was calculated in each site of the pair. Distribution of these relative abundances of shared ASVs between two sites is presented as boxplot. For each pair of site and each time point, the left boxplot correspond to the relative abundance of shared ASVs in the site mentioned in line and the right boxplot correspond to the relative abundance of shared ASVs in the site mentioned in column. Boxplot median and colored square represent the median and mean relative abundances of shared ASVs between the two sites respectively.

**Additional file 9**. **Total number of shared ASVs between three sites or four sites at the animal level.** Shared ASVs between three or four sites are identified for each animal and each time point. A total of 635 combinationswere obtained, corresponding to 43 animals and 151 distinct ASVs.

**Additional file 10**. List of ASVs shared between 3 or 4 anatomic sites in more than 10 animals

**Additional file 11**. **Beta dispersion results.** Results of the permutations tests of homogeneity of beta-dispersion across sitesor along timefor the UniFrac and Bray–Curtis distances.

**Additional file 12**. MDS plots of the site microbiota at different time points, for the Bray–Curtis distance. Same legend as in Fig. 4. Although the PERMANOVA *P* values can be anticonservative due to differences in dispersion, the microbiota differs across sites.

**Additional file 13**. **Kitome ASVs.** Short name, sequence, taxonomic classification and total read number in the dataset of the kitome ASVs.

## Data Availability

The datasets generated and/or analyzed during the current study are available in the Sequence Read Archive of the National Center for Biotechnology Information under the accession number PRJNA 875059.

## Abbreviations

SM: Susceptibility to mastitis
CM: Clinical mastitis
BC: Body condition
ASV: Amplicon sequence variants
MSD: Multi-dimensional scaling
ARI: Adjusted Rand Index
OTU: Operational Taxonomic Units
1W: One week before parturition
1 M, 3 M, 7 M: 1, 3 and 7 months post parturition
