# Supplementary material for: Microbiota members from body sites of dairy cows are largely shared within individual hosts throughout lactation but sharing is limited in the herd
Source: Anim Microbiome. 2023 Jun 12;5:32. doi: 10.1186/s42523-023-00252-w (PMC10262541; doi:10.1186/s42523-023-00252-w)
Supplement: Supplementary file 1 — Additional file 1. Animal description and associated metadata. [file 42523_2023_252_MOESM1_ESM.pdf]

Additional file 1. Animal description and associated metadata

animal ID	group of animals	SM_score[1]	BC_score[2]	calving date	housing[3] -1S	housing[3] 1M	housing[3] 3M	housing[3] 7M	diet[4] -1S	diet[4] 1M	diet[4] 3M	diet[4] 7M	sampling date and time[5] -1S	sampling date and time[5] 1M	sampling date and time[5] 3M	sampling date and time[5] 7M
1	S1	1,70	0,90	30/10/2015	stab_1	stab_2	stab_2	grazing	R1	R2	R2	grazing	26/10/2015 afternoon	01/12/2015 morning	02/02/2016 morning	30/05/2016 morning
2	S1	-1,50	0,30	30/10/2015												
3	S1	-1,60	-1,20	30/10/2015												
4	S1	-3,70	0,40	29/10/2015												
5	S1	-0,50	-0,30	01/11/2015												
6	S1	0,00	-0,60	01/11/2015												
7	S1	0,00	0,50	01/11/2015												
8	S1	0,60	1,10	30/10/2015												
9	S1	1,00	1,60	30/10/2015												
10	S1	0,90	0,90	30/10/2015												
11	S1	0,50	0,60	30/10/2015												
12	S1	-0,20	1,10	30/10/2015												
13	S1	-0,20	1,40	30/10/2015												
14	S1	0,40	1,10	29/10/2015												
15	S1	0,60	2,00	30/10/2015												
16	S1	-0,40	-0,80	30/10/2015												
17	S1	1,90	2,20	30/10/2015												
18	S1	-0,30	0,50	01/11/2015												
19	S2	-1,50	-1,00	21/11/2015	stab_1 (grazing before parturition)	stab_3	stab_3	grazing	R2	R2	R2	grazing	10/11/2015 morning	21/12/2015 afternoon	23/02/2016 morning	21/06/2016 morning
20	S2	-1,80	0,20	29/11/2015												
21	S2	-0,20	0,30	20/11/2015												
22	S2	-0,60	-0,30	22/11/2015												
23	S2	-1,50	0,10	20/11/2015												
24	S2	0,00	0,20	22/11/2015												
25	S2	0,30	0,90	22/11/2015												
26	S2	-0,80	1,30	22/11/2015												
27	S2	-0,10	-0,50	22/11/2015												
28	S2	0,80	-0,20	22/11/2015												
29	S2	0,80	0,20	21/11/2015												
30	S2	0,00	0,20	20/11/2015												
31	S2	1,50	0,50	22/11/2015												
32	S2	1,80	0,70	21/11/2015												
45	S2	-0,30	1,10	24/11/2015												
33	S3	-0,80	-0,80	04/01/2016	stab_1 (grazing before parturition)	stab_3	stab_3	grazing	R3	R2	R2	grazing_ corn	21/12/2015 afternoon	01/02/2016 morning	31/03/2016 morning	25/07/2016 morning
34	S3	-0,70	0,10	02/01/2016												
35	S3	0,80	-0,20	10/01/2016												
36	S3	0,10	0,10	07/01/2016												
37	S3	-1,10	-0,70	03/01/2016												
38	S3	0,30	0,90	03/01/2016												
39	S3	0,50	0,50	02/01/2016												
40	S3	0,50	0,40	02/01/2016												
41	S3	2,60	0,70	10/01/2016												
42	S3	0,80	-0,60	02/01/2016												
43	S3	-0,30	0,80	04/01/2016												
44	S3	0,30	1,20	10/01/2016												

[1] SM_score : score of susceptibility to mastitis estimated from cow genotype: negative score correspond to animals with a higher susceptibility to mastitis, positive score correspond to animals with a higher resistance to mastitis

[2] BC_score: score of body condition estimated from cow genotype: negative score correspond to animals with lower body condition, positive score correspond to animals with higher body condition

3 Housing : stab_1, stab_2, stab_3 correspond to 3 different stabulations (deep litter housing with daily mulching)

4 diet : animals were fed with 19 kg of DM/d of either ration R1 (55% corn silage, 25% wrapped bale silage, 12% rapeseed, 8% energy concentrate, 250g minerals), ration R2 (55% corn silage, 25% grass silage, 12% rapeseed, 8% energy concentrate, 250g minerals) or ration R3 (50% wrapped bale silage, 50% hay), or grazing or grazing combined with corn silage (4 4 kg of DM/d)

[5] sampling time: the date and time of sampling are indicated. Sampling of vaginal, oral and nasal cavities wer performed either following morning milking or in the afternoon, prior to the evening milking.
