# Supplementary figures and images for: Microbiota members from body sites of dairy cows are largely shared within individual hosts throughout lactation but sharing is limited in the herd
Source: Anim Microbiome. 2023 Jun 12;5:32. doi: 10.1186/s42523-023-00252-w (PMC10262541; doi:10.1186/s42523-023-00252-w)

**A**

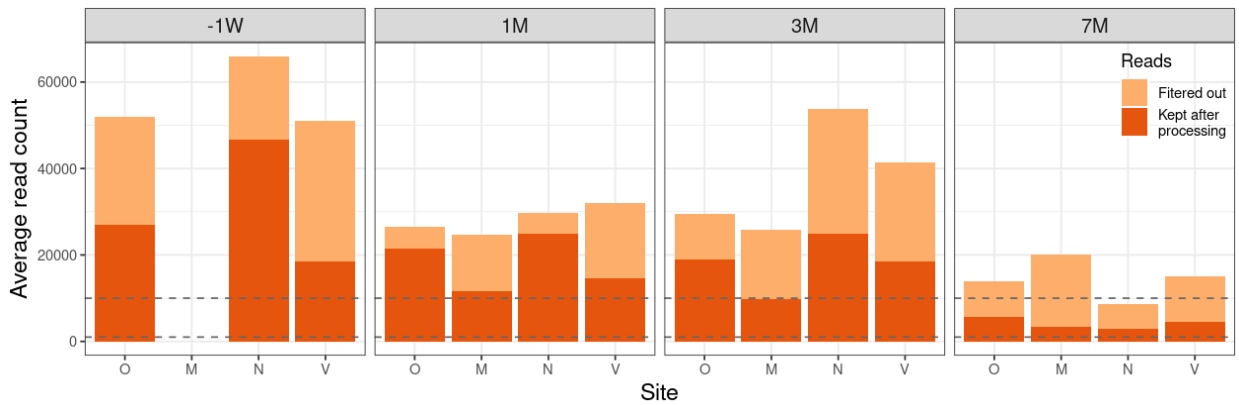

**B**

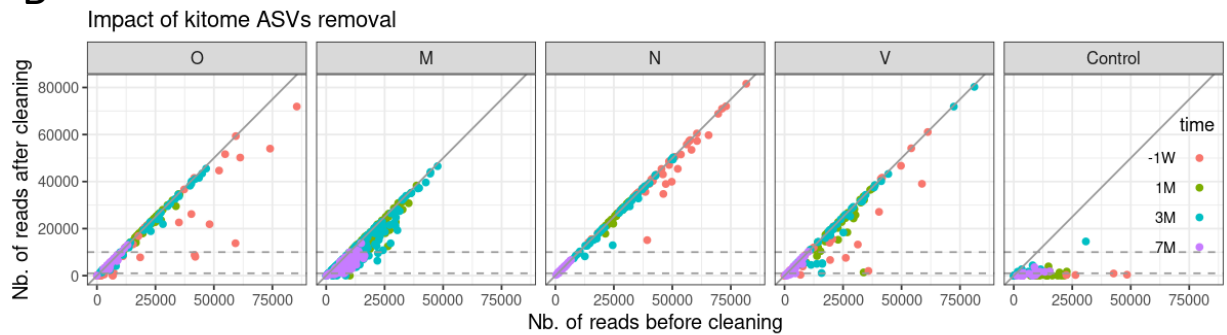

**C**

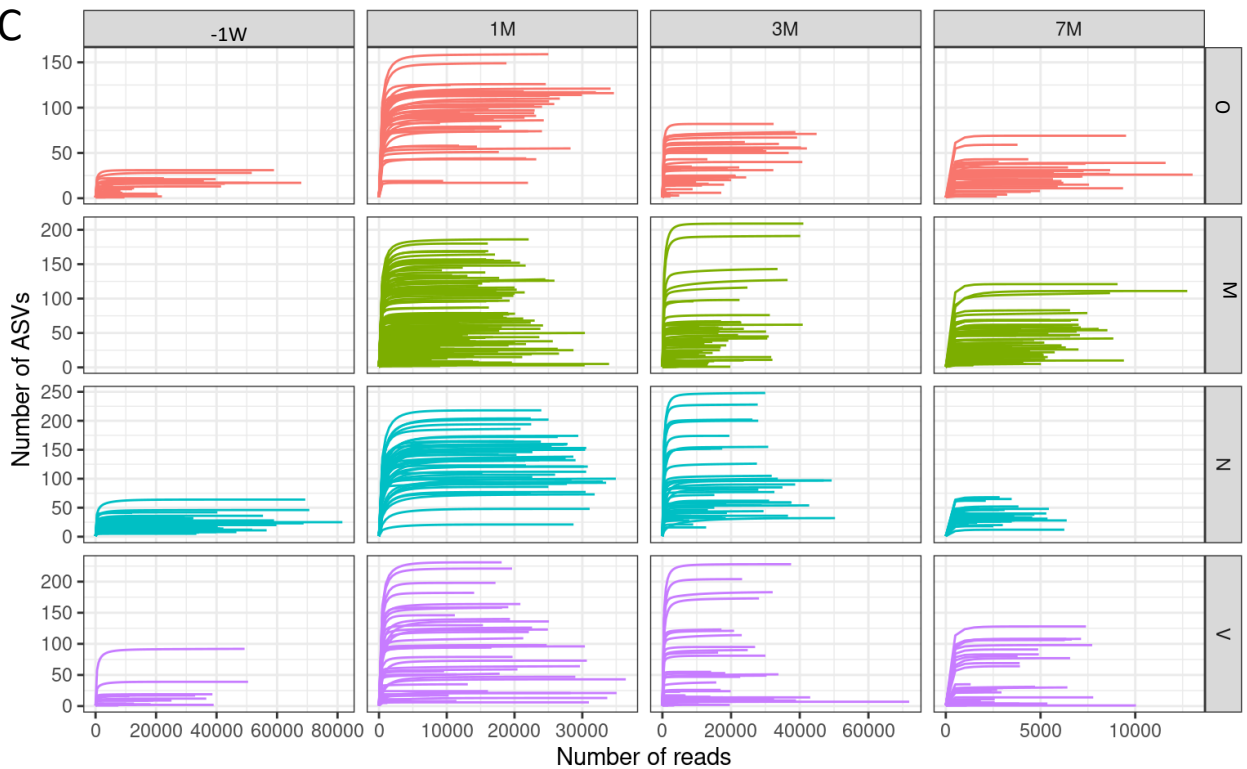

Supplement: Supplementary file 2 — Additional file 2. Sequencing depth. A. Average sequencing depth in each anatomic site and each time point, before processingand following pre-processing and filtration steps. Dotted lines indicate an average read count of 1000 and 10,000 reads. B. Impact of the kitome ASVs removal on the number of reads. Dotted lines indicate an average read count of 1000 and 10,000 reads. C. rarefaction curves on processed data. [file 42523_2023_252_MOESM2_ESM.pdf]

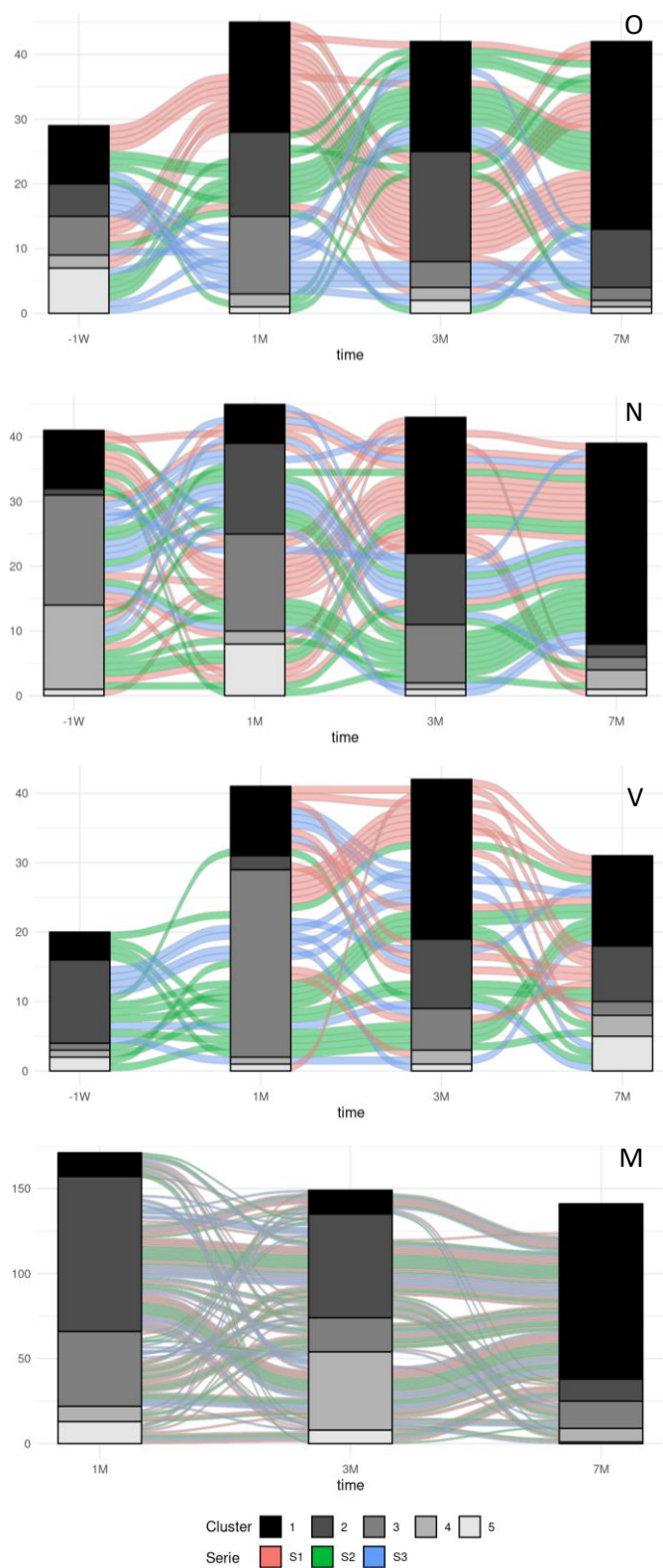

|               | Oral | Nasal | Vaginal | Milk |
|---------------|------|-------|---------|------|
| -1W versus 1M | 0.12 | 0.07  | -0.13   |      |
| 1M versus 3M  | 0.31 | 0.04  | 0.01    | 0.07 |
| 3M versus 7M  | 0.17 | -0.05 | 0.01    | 0.05 |

Supplement: Supplementary file 5 — Additional file 5. Alluvial plot of cows across microbial clusters. The alluvial plot shows how each cowflows across clusters. Ribbons departing from the same cluster and splitting towards several clusters means that the clusters are not conserved across times. Clustering of the microbiota at each site and time point was performed using hierarchical clustering and cutting the dendrogram to have 5 clusters. This number of clusters was chosen so that cluster size allowed pointing out differences in cluster composition. For each site, the distributions of cows into clusters were compared between consecutive time points using the ARI to assess the temporal stability of the clusters. Positive yet very low ARI values, indicative of limited stability, were observed for the nasal microbiota between 1 week before parturition and 1 M and 3 M, and for the milk microbiota between all-time points, while no stability was observed for vaginal microbiota. ARI values were higher for the oral microbiota at all time points, with the highest valueobserved between 1 and 3 M as well as between 3 and 7 M. This stability of the clusters across time points was likely due to a “group of animals” effect, and thus to the environmental conditions of each group of animals, as highlighted by ribbons with the same colormoving together across clusters. Table indicates the Adjusted Rand Indexof animal clustering across time. 1 corresponds to equal clustering whereas 0 is the score expected for two random clusterings. [file 42523_2023_252_MOESM5_ESM.pdf]

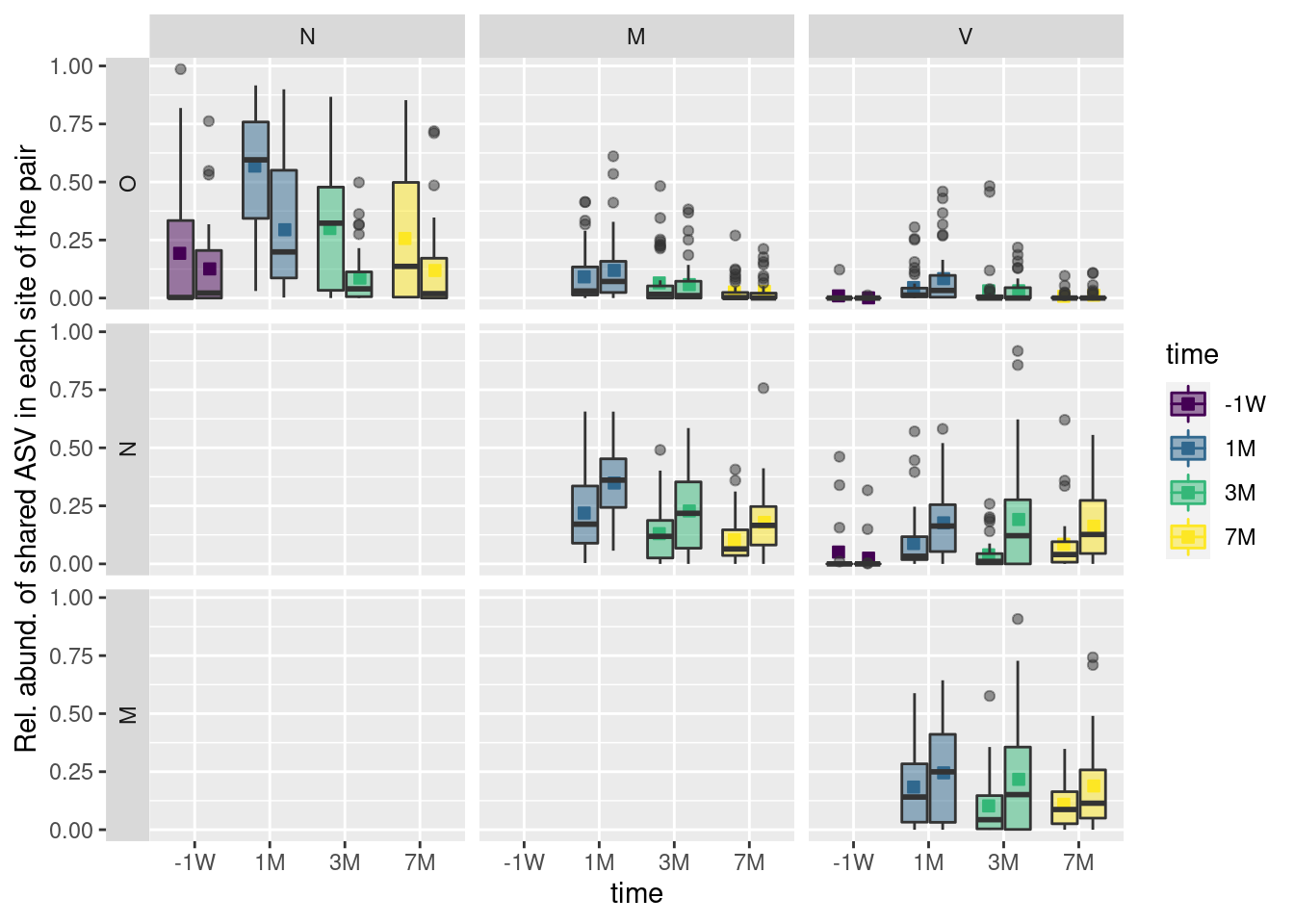

Supplement: Supplementary file 8 — Additional file 8. Relative abundance of shared ASVs between the different anatomic sites at the animal level. For each animal and each time point, the total relative abundance of shared ASVs between the two sites was calculated in each site of the pair. Distribution of these relative abundances of shared ASVs between two sites is presented as boxplot. For each pair of site and each time point, the left boxplot correspond to the relative abundance of shared ASVs in the site mentioned in line and the right boxplot correspond to the relative abundance of shared ASVs in the site mentioned in column. Boxplot median and colored square represent the median and mean relative abundances of shared ASVs between the two sites respectively. [file 42523_2023_252_MOESM8_ESM.png]

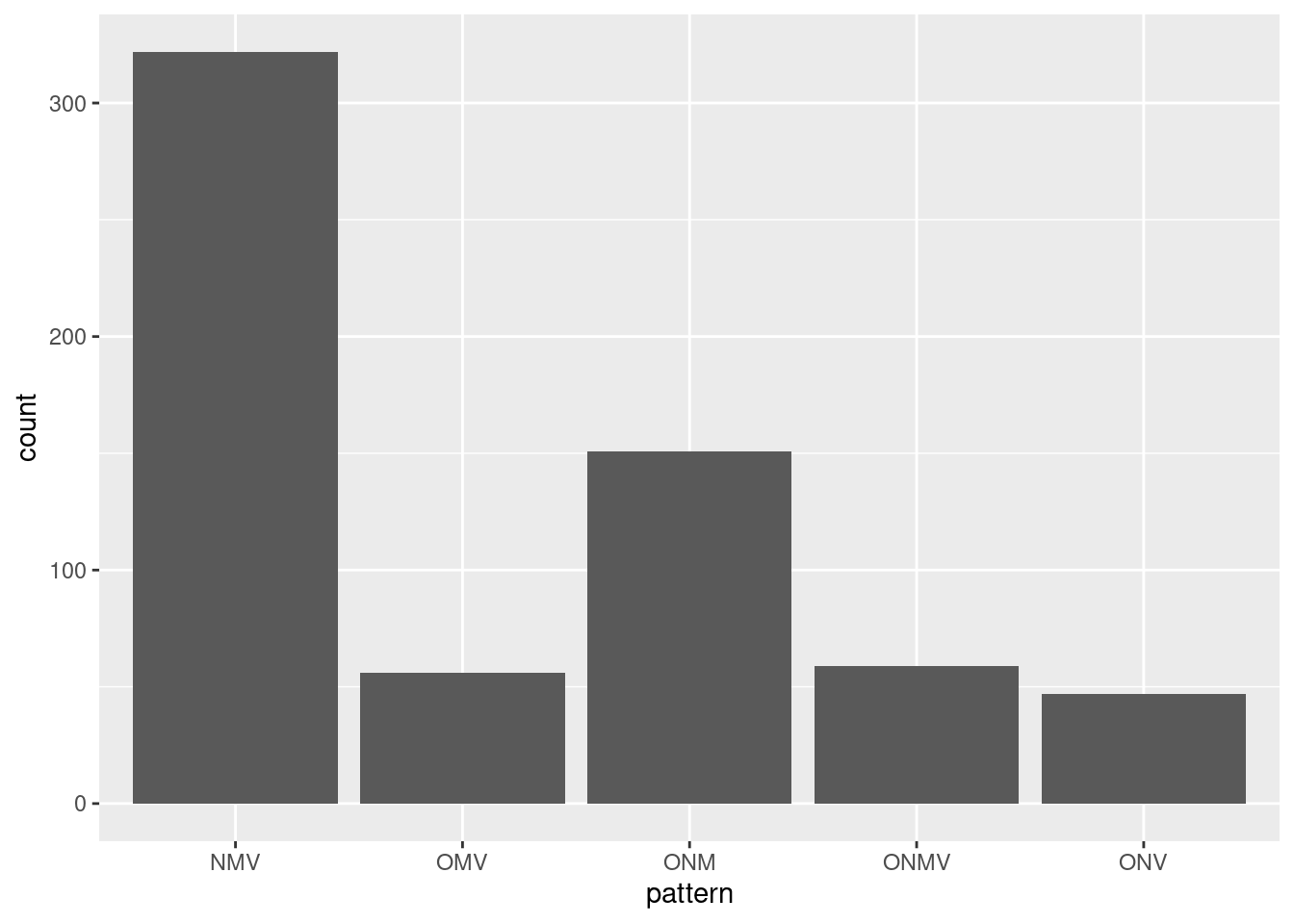

Supplement: Supplementary file 9 — Additional file 9. Total number of shared ASVs between three sites or four sites at the animal level. Shared ASVs between three or four sites are identified for each animal and each time point. A total of 635 combinationswere obtained, corresponding to 43 animals and 151 distinct ASVs. [file 42523_2023_252_MOESM9_ESM.png]

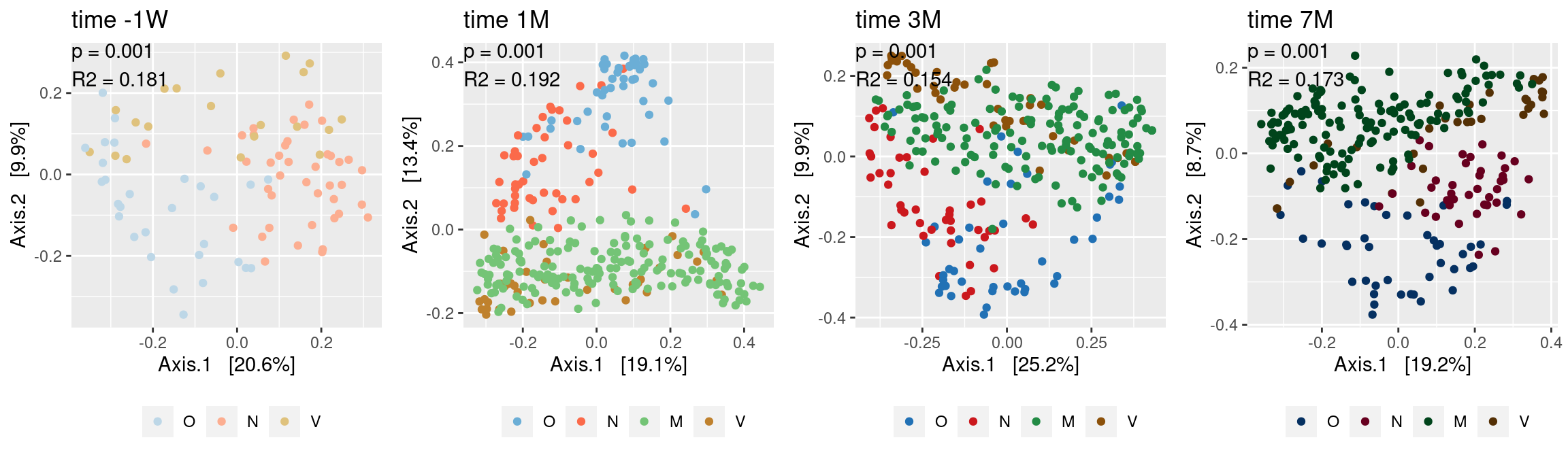

Supplement: Supplementary file 12 — Additional file 12. MDS plots of the site microbiota at different time points, for the Bray–Curtis distance. Same legend as in Fig. 4. Although the PERMANOVA P values can be anticonservative due to differences in dispersion, the microbiota differs across sites. [file 42523_2023_252_MOESM12_ESM.png]
