# Supplementary material for: Microbiota members from body sites of dairy cows are largely shared within individual hosts throughout lactation but sharing is limited in the herd
Source: Anim Microbiome. 2023 Jun 12;5:32. doi: 10.1186/s42523-023-00252-w (PMC10262541; doi:10.1186/s42523-023-00252-w)
Supplement: Supplementary file 3 — Additional file 3. Dominant genera in oral, nasal, vaginal and milk microbiota. For each anatomic site, the 25 more abundant genera at each sampling time are listed with their rank, mean abundance and prevalence at each sampling time. In Bold: mean abundances > 5%, in blue: 25 dominant genera for each site at each time point, and corresponding prevalence. [file 42523_2023_252_MOESM3_ESM.pdf]

Additional file 3. Dominant genera in oral, nasal, vaginal and milk microbiota. For each anatomic site, the 25 more abundant genera at each sampling time are listed with their rank, mean abundance and prevalence at each sampling time. In Bold: mean abundances >5%, in blue: 25 dominant genera for each site at each time point, and corresponding prevalence

						rank[(1)]				mean abundance (%)				prevalence (%)			
**Site**	**Phylum**	**Class**	**Order**	**Family**	**Genus**	**-1W**	**1M**	**3M**	**7M**	**-1W**	**1M**	**3M**	**7M**	**-1W**	**1M**	**3M**	**7M**
		Actinobacteria	Bifidobacteriales	Bifidobacteriaceae	Bifidobacterium	87	**25**	45	147	0,00	0,452	0,231	0,0041	0,00	40	16,7	2,38
		Actinobacteria	Corynebacteriales	Corynebacteriaceae	Corynebacterium 1	**17**	**11**	26	26	1,71	1,58	0,794	0,455	10,3	64,4	33,3	28,6
		Actinobacteria	Micrococcales	Brevibacteriaceae	Brevibacterium	**4**	49	66	118	**8,20**	0,158	0,132	0,0214	31	37,8	14,3	4,76
	Actinobacteria	Actinobacteria	Micrococcales	Microbacteriaceae	Clavibacter	**8**	94	89	101	3,45	0,0495	0,0581	0,044	3,45	6,67	14,3	2,38
		Actinobacteria	Micrococcales	Microbacteriaceae	Multi-affiliation	**25**	44	**17**	168	0,417	0,195	1,43	0,00	6,90	24,4	35,7	0,00
		Actinobacteria	Micrococcales	Micrococcaceae	Kocuria	**13**	106	84	131	2,41	0,0403	0,0713	0,0115	20,7	8,89	4,76	2,38
		Actinobacteria	Micrococcales	Micrococcaceae	Rothia	**15**	68	65	59	2,18	0,0931	0,136	0,157	10,3	13,3	9,52	9,52
		Bacteroidia	Bacteroidales	Prevotellaceae	Prevotella 1	54	201	137	**8**	0,0535	0,00204	0,002	4,90	3,45	4,44	2,38	45,2
		Bacteroidia	Bacteroidales	Prevotellaceae	Prevotellaceae NK3B31 group	135	245	174	**16**	0,00	0,00	0,00	0,714	0,00	0,00	0,00	14,3
		Bacteroidia	Cytophagales	Spirosomaceae	Pseudarcicella	55	54	**18**	201	0,0508	0,137	1,24	0,00	6,90	24,4	2,38	0,00
	Bacteroidetes	Bacteroidia	Flavobacteriales	Weeksellaceae	Bergeyella	37	67	46	**12**	0,223	0,0944	0,218	1,62	10,3	13,3	9,52	42,9
		Bacteroidia	Flavobacteriales	Weeksellaceae	Chryseobacterium	151	**16**	76	55	0,00	1,21	0,0909	0,167	0,00	86,7	16,7	7,14
		Bacteroidia	Flavobacteriales	Weeksellaceae	Empedobacter	152	**20**	185	120	0,00	0,708	0,00	0,0188	0,00	64,4	0,00	2,38
		Bacteroidia	Sphingobacteriales	Sphingobacteriaceae	Sphingobacterium	83	**14**	43	67	0,00029	1,26	0,248	0,112	3,45	86,7	19	7,14
		Bacilli	Bacillales	Bacillaceae	Bacillus	61	**10**	77	57	0,0178	1,61	0,087	0,164	3,45	77,8	14,3	16,7
		Bacilli	Bacillales	Staphylococcaceae	Staphylococcus	**9**	64	111	27	3,37	0,102	0,0205	0,451	20,7	28,9	9,52	14,3
		Bacilli	Lactobacillales	Lactobacillaceae	Lactobacillus	**1**	**1**	**1**	**1**	**20,4**	**24,6**	**20,8**	**12**	55,2	100	85,7	28,6
		Bacilli	Lactobacillales	Lactobacillaceae	Multi-affiliation	38	**24**	**24**	60	0,221	0,523	0,878	0,154	17,2	88,9	42,9	16,7
		Bacilli	Lactobacillales	Lactobacillaceae	Pediococcus	**14**	**15**	**8**	**25**	2,33	1,23	2,45	0,461	20,7	88,9	57,1	11,9
		Bacilli	Lactobacillales	Leuconostocaceae	Leuconostoc	**6**	**3**	**2**	51	4,88	**5,09**	**11,3**	0,194	37,9	91,1	69	7,14
		Bacilli	Lactobacillales	Leuconostocaceae	Weissella	**10**	**17**	**6**	98	3,22	1,06	3,37	0,0461	24,1	88,9	64,3	4,76
		Bacilli	Lactobacillales	Streptococcaceae	Streptococcus	**21**	76	47	**7**	0,756	0,0802	0,216	**5,02**	3,45	22,2	11,9	64,3
	Firmicutes	Clostridia	Clostridiales	Clostridiaceae 1	Clostridium sensu stricto 12	**23**	75	73	91	0,654	0,0804	0,104	0,0576	6,90	48,9	14,3	9,52
		Clostridia	Clostridiales	Clostridiaceae 1	Multi-affiliation	74	**23**	**23**	56	0,00187	0,547	0,951	0,166	3,45	60	35,7	11,9
		Clostridia	Clostridiales	Lachnospiraceae	[Eubacterium] cellulosolvens group	206	280	227	**15**	0,00	0,00	0,00	0,797	0,00	0,00	0,00	11,9
		Clostridia	Clostridiales	Lachnospiraceae	unknown genus	237	126	63	**23**	0,00	0,0237	0,143	0,472	0,00	11,1	7,14	9,52
		Clostridia	Clostridiales	Multi-affiliation	Multi-affiliation	30	28	**15**	**18**	0,352	0,407	1,45	0,593	17,2	48,9	33,3	23,8
		Clostridia	Clostridiales	Ruminococcaceae	Ruminococcaceae NK4A214 group	250	175	261	**21**	0,00	0,00447	0,00	0,55	0,00	4,44	0,00	16,7
		Clostridia	Clostridiales	Ruminococcaceae	Ruminococcaceae UCG-005	35	41	**14**	**19**	0,23	0,204	1,57	0,574	3,45	33,3	28,6	28,6
Oral		Clostridia	Clostridiales	Ruminococcaceae	Ruminococcus 1	255	304	264	**20**	0,00	0,00	0,00	0,558	0,00	0,00	0,00	14,3
		Clostridia	Clostridiales	Ruminococcaceae	Saccharofermentans	257	197	265	**22**	0,00	0,00248	0,00	0,525	0,00	2,22	0,00	11,9
		Alphaproteobacteria	Acetobacterales	Acetobacteraceae	Acetobacter	**7**	**2**	**3**	**5**	4,29	**21**	**8,93**	**8,21**	27,6	100	69	26,2
		Alphaproteobacteria	Rhizobiales	Rhizobiaceae	Allorhizobium-Neorhizobium-Pararhizobium-Rhizobium	48	40	**22**	104	0,0847	0,207	0,964	0,0384	13,8	53,3	40,5	2,38
		Alphaproteobacteria	Rhodobacterales	Rhodobacteraceae	Paracoccus	**19**	72	57	38	0,979	0,0868	0,158	0,26	3,45	11,1	7,14	7,14
		Alphaproteobacteria	Sphingomonadales	Sphingomonadaceae	Sphingomonas	**20**	**22**	**25**	**14**	0,768	0,557	0,816	0,971	13,8	64,4	19	16,7
		Alphaproteobacteria	Sphingomonadales	Sphingomonadaceae	unknown genus	**22**	321	296	142	0,658	0,00	0,00	0,00563	3,45	0,00	0,00	2,38
		Gammaproteobacteria	Betaproteobacteriales	Burkholderiaceae	Comamonas	304	**7**	38	73	0,00	3,65	0,429	0,0844	0,00	73,3	26,2	9,52
		Gammaproteobacteria	Betaproteobacteriales	Burkholderiaceae	Ralstonia	**12**	157	28	318	2,59	0,0123	0,697	0,00	10,3	2,22	11,9	0,00
		Gammaproteobacteria	Betaproteobacteriales	Neisseriaceae	Alysiella	**3**	329	318	**9**	**8,40**	0,00	0,00	3,36	27,6	0,00	0,00	52,4
		Gammaproteobacteria	Enterobacteriales	Enterobacteriaceae	Hafnia-Obesumbacterium	52	**18**	**10**	324	0,0616	1,06	2,07	0,00	6,90	77,8	50	0,00
	Proteobacteria	Gammaproteobacteria	Enterobacteriales	Enterobacteriaceae	Multi-affiliation	57	**9**	**12**	70	0,0377	2,03	1,95	0,0961	6,90	88,9	54,8	7,14
		Gammaproteobacteria	Enterobacteriales	Enterobacteriaceae	Pantoea	**16**	47	**16**	81	1,82	0,167	1,45	0,0712	3,45	24,4	31	2,38
		Gammaproteobacteria	Enterobacteriales	Enterobacteriaceae	Proteus	324	**19**	327	327	0,00	0,736	0,00	0,00	0,00	31,1	0,00	0,00
		Gammaproteobacteria	Pasteurellales	Pasteurellaceae	Bibersteinia	**2**	**5**	**5**	**3**	**9,34**	3,84	3,98	**10,8**	37,9	46,7	42,9	83,3
		Gammaproteobacteria	Pasteurellales	Pasteurellaceae	Haemophilus	**5**	38	33	**2**	**6,05**	0,251	0,591	**11,9**	27,6	24,4	14,3	73,8
		Gammaproteobacteria	Pasteurellales	Pasteurellaceae	Mannheimia	33	219	330	**11**	0,296	0,00058	0,00	2,18	3,45	2,22	0,00	40,5
		Gammaproteobacteria	Pasteurellales	Pasteurellaceae	unknown genus	27	59	50	**17**	0,397	0,118	0,196	0,602	13,8	15,6	9,52	33,3
		Gammaproteobacteria	Pseudomonadales	Moraxellaceae	Acinetobacter	64	**21**	**7**	**6**	0,00949	0,662	3,08	**5,13**	3,45	84,4	52,4	64,3
		Gammaproteobacteria	Pseudomonadales	Moraxellaceae	Moraxella	29	31	**11**	**4**	0,369	0,329	2,00	**10,4**	17,2	13,3	16,7	76,2
		Gammaproteobacteria	Pseudomonadales	Pseudomonadaceae	Pseudomonas	34	**12**	**19**	145	0,239	1,44	1,21	0,0045	3,45	88,9	45,2	2,38
		Gammaproteobacteria	Xanthomonadales	Xanthomonadaceae	SN8	68	**13**	31	337	0,00597	1,41	0,659	0,00	3,45	77,8	16,7	0,00
		Gammaproteobacteria	Xanthomonadales	Xanthomonadaceae	Stenotrophomonas	337	**6**	101	144	0,00	3,81	0,0306	0,00525	0,00	75,6	7,14	2,38
	Verrucomicrobia	Verrucomicrobiae	Verrucomicrobiales	Verrucomicrobiaceae	Prosthecobacter	**18**	343	56	343	1,28	0,00	0,164	0,00	10,3	0,00	7,14	0,00
	Cyanobacteria	Oxyphotobacteria	Chloroplast	unknown family	unknown genus	**24**	**4**	**9**	**13**	0,43	3,98	2,35	1,35	17,2	71,1	35,7	31
	Dependentiae	Babeliae	Babeliales	Babeliaceae	unknown genus	162	260	**21**	213	0,00	0,00	1,04	0,00	0,00	0,00	7,14	0,00
	Euryarchaeota	Methanobacteria	Methanobacteriales	Methanobacteriaceae	Methanobrevibacter	63	140	**20**	48	0,00978	0,0166	1,14	0,211	3,45	15,6	14,3	19
	Fibrobacteres	Fibrobacteria	Fibrobacterales	Fibrobacteraceae	Fibrobacter	81	262	196	**24**	0,00053	0,00	0,00	0,466	3,45	0,00	0,00	19
	Patescibacteria	Parcubacteria	Candidatus Zambryskibacteria	unknown family	unknown genus	51	192	**13**	65	0,0635	0,00273	1,69	0,122	3,45	2,22	2,38	9,52
	Multi-affiliation	Multi-affiliation	Multi-affiliation	Multi-affiliation	Multi-affiliation	**11**	**8**	**4**	**10**	3,22	3,63	5,05	2,92	24,1	93,3	61,9	28,6

Site	Phylum	Class	Order	Family	Genus	rank[1] -1W	1M	3M	7M	mean abundance (%) -1W	1M	3M	7M	prevalence (%) -1W	1M	3M	7M
		Actinobacteria	Bifidobacteriales	Bifidobacteriaceae	Bifidobacterium	11	20	38	100	1,98	0,882	0,515	0,0753	24,4	97,8	58,1	15,4
		Actinobacteria	Corynebacteriales	Corynebacteriaceae	Corynebacterium 1	7	4	10	17	2,57	**6,32**	2,79	1,17	56,1	100	76,7	59
		Actinobacteria	Micrococcales	Brevibacteriaceae	Brevibacterium	5	34	25	21	4,76	0,396	1,01	0,914	29,3	77,8	46,5	43,6
	Actinobacteria	Actinobacteria	Micrococcales	Microbacteriaceae	Candidatus Limnoluna	2	18	21	2	**8,19**	0,97	1,24	**8,62**	46,3	55,6	34,9	53,8
		Actinobacteria	Micrococcales	Microbacteriaceae	Multi-affiliation	17	12	26	37	1,15	1,41	0,967	0,425	9,76	71,1	74,4	25,6
		Actinobacteria	Micrococcales	Micrococcaceae	Glutamicibacter	33	69	14	54	0,463	0,163	2,10	0,221	17,1	42,2	53,5	15,4
		Actinobacteria	Micrococcales	Micrococcaceae	Kocuria	9	46	29	15	2,06	0,279	0,774	1,51	24,4	55,6	39,5	41
		Actinobacteria	Micrococcales	Micrococcaceae	Micrococcus	122	21	92	62	0,00969	0,754	0,119	0,184	2,44	82,2	16,3	10,3
		Bacteroidia	Bacteroidales	Bacteroidaceae	Bacteroides	26	63	20	38	0,601	0,187	1,25	0,421	17,1	71,1	62,8	38,5
		Bacteroidia	Bacteroidales	Prevotellaceae	Prevotella 1	22	147	124	13	0,667	0,0213	0,0641	1,67	12,2	24,4	16,3	30,8
	Bacteroidetes	Bacteroidia	Chitinophagales	Chitinophagaceae	Filobacterium	1	2	4	7	**28,7**	**10,8**	4,93	4,62	95,1	97,8	74,4	76,9
		Bacteroidia	Flavobacteriales	Weeksellaceae	Chryseobacterium	193	36	113	16	0,00	0,379	0,0792	1,49	0,00	88,9	23,3	5,13
		Bacteroidia	Sphingobacteriales	Sphingobacteriaceae	Sphingobacterium	196	25	77	229	0,00	0,593	0,168	0,00	0,00	77,8	27,9	0,00
		Bacilli	Bacillales	Planococcaceae	Kurthia	35	22	36	70	0,438	0,689	0,58	0,14	24,4	77,8	53,5	2,56
		Bacilli	Bacillales	Planococcaceae	Multi-affiliation	42	13	17	20	0,357	1,35	1,90	0,935	36,6	97,8	76,7	56,4
		Bacilli	Bacillales	Staphylococcaceae	Staphylococcus	3	17	15	18	**6,43**	0,999	2,09	1,15	65,9	86,7	76,7	56,4
		Bacilli	Lactobacillales	Aerococcaceae	unknown genus	24	191	131	160	0,659	0,00946	0,0498	0,0149	12,2	4,44	9,30	2,56
		Bacilli	Lactobacillales	Lactobacillaceae	Lactobacillus	4	3	12	6	4,85	**10,6**	2,41	4,71	58,5	97,8	74,4	33,3
		Bacilli	Lactobacillales	Streptococcaceae	Lactococcus	19	160	117	172	0,782	0,0178	0,0748	0,00986	2,44	6,67	23,3	2,56
		Bacilli	Lactobacillales	Streptococcaceae	Streptococcus	13	1	1	3	1,87	**16,5**	**11,3**	**6,10**	26,8	82,2	81,4	61,5
	Firmicutes																
		Clostridia	Clostridiales	Lachnospiraceae	[Eubacterium] cellulosolvens group	77	294	273	22	0,103	0,00	0,00	0,905	2,44	0,00	0,00	28,2
		Clostridia	Clostridiales	Lachnospiraceae	Lachnospiraceae NK3A20 group	14	27	18	29	1,81	0,56	1,41	0,607	41,5	86,7	72,1	23,1
Nasal		Clostridia	Clostridiales	Multi-affiliation	Multi-affiliation	12	10	13	5	1,91	2,33	2,12	4,79	48,8	100	69,8	89,7
		Clostridia	Clostridiales	Peptostreptococcaceae	Multi-affiliation	16	11	19	12	1,23	1,76	1,36	1,89	48,8	97,8	62,8	74,4
		Clostridia	Clostridiales	Peptostreptococcaceae	Romboutsia	8	48	47	50	2,14	0,271	0,304	0,248	12,2	68,9	34,9	20,5
		Clostridia	Clostridiales	Ruminococcaceae	Multi-affiliation	20	153	105	106	0,739	0,019	0,0893	0,0661	2,44	13,3	14	7,69
		Clostridia	Clostridiales	Ruminococcaceae	Ruminococcaceae UCG-005	15	23	11	9	1,58	0,678	2,75	3,13	43,9	86,7	69,8	69,2
		Erysipelotrichia	Erysipelotrichales	Erysipelotrichaceae	Turicibacter	45	24	33	27	0,334	0,641	0,679	0,651	29,3	91,1	60,5	53,8
		Alphaproteobacteria	Acetobacterales	Acetobacteraceae	Acetobacter	55	5	30	10	0,215	**5,54**	0,752	3,01	17,1	82,2	44,2	28,2
		Alphaproteobacteria	Rhizobiales	Rhizobiaceae	Allorhizobium-Neorhizobium-Pararhizobium-Rhizobium	21	58	44	123	0,684	0,213	0,339	0,0434	17,1	64,4	51,2	5,13
		Alphaproteobacteria	Sphingomonadales	Sphingomonadaceae	Sphingomonas	23	7	8	28	0,665	2,92	3,34	0,627	41,5	95,6	88,4	46,2
		Gammaproteobacteria	Alteromonadales	Pseudoalteromonadaceae	Pseudoalteromonas	43	323	2	307	0,34	0,00	**6,27**	0,00	2,44	0,00	32,6	0,00
		Gammaproteobacteria	Betaproteobacteriales	Burkholderiaceae	Massilia	31	15	16	96	0,545	1,07	2,05	0,0803	17,1	73,3	72,1	10,3
		Gammaproteobacteria	Multi-affiliation	Multi-affiliation	Multi-affiliation	95	85	9	24	0,0552	0,101	2,90	0,794	4,88	48,9	60,5	30,8
	Proteobacteria	Gammaproteobacteria	Oceanospirillales	Halomonadaceae	Marinospirillum	85	224	331	11	0,08	0,00436	0,00	1,89	9,76	4,44	0,00	48,7
		Gammaproteobacteria	Pasteurellales	Pasteurellaceae	Mannheimia	329	8	6	26	0,00	2,69	4,05	0,681	0,00	22,2	34,9	12,8
		Gammaproteobacteria	Pasteurellales	Pasteurellaceae	Pasteurella	18	31	22	14	0,99	0,483	1,21	1,60	9,76	11,1	6,98	5,13
		Gammaproteobacteria	Pseudomonadales	Moraxellaceae	Acinetobacter	27	16	24	19	0,583	1,04	1,10	0,963	43,9	95,6	79,1	61,5
		Gammaproteobacteria	Pseudomonadales	Moraxellaceae	Moraxella	10	9	5	1	2,03	2,39	4,24	**18,1**	19,5	35,6	48,8	74,4
		Gammaproteobacteria	Pseudomonadales	Moraxellaceae	Psychrobacter	34	14	3	58	0,452	1,08	**5,09**	0,208	12,2	71,1	53,5	17,9
		Gammaproteobacteria	Pseudomonadales	Pseudomonadaceae	Pseudomonas	39	19	7	105	0,376	0,92	3,62	0,0699	14,6	84,4	81,4	7,69
		Gammaproteobacteria	Xanthomonadales	Xanthomonadaceae	Luteimonas	335	342	337	23	0,00	0,00	0,00	0,876	0,00	0,00	0,00	7,69
	Tenericutes	Mollicutes	Mycoplasmatales	Mycoplasmataceae	Mycoplasma	6	6	23	8	3,87	**5,41**	1,13	3,58	61	91,1	53,5	74,4
	Cyanobacteria	Oxyphotobacteria	Chloroplast	unknown family	unknown genus	75	68	32	25	0,107	0,171	0,683	0,702	12,2	42,2	23,3	15,4
	Fusobacteria	Fusobacteriia	Fusobacteriales	Fusobacteriaceae	Fusobacterium	25	87	102	286	0,64	0,0982	0,0925	0,00	2,44	33,3	2,33	0,00
	Multi-affiliation	Multi-affiliation	Multi-affiliation	Multi-affiliation	Multi-affiliation	124	59	27	4	0,00852	0,209	0,961	4,87	9,76	64,4	46,5	71,8

						rank(1)				mean abundance (%)				prevalence (%)			
Site	Phylum	Class	Order	Family	Genus	-1W	1M	3M	7M	-1W	1M	3M	7M	-1W	1M	3M	7M
		Actinobacteria	Actinomycetales	Actinomycetaceae	Trueperella	32	10	56	125	0,537	2,09	0,284	0,00	10	41,5	7,14	0
		Actinobacteria	Corynebacteriales	Corynebacteriaceae	Corynebacterium	14	22	81	8	2,13	1,10	0,137	2,75	25	43,9	14,3	61,3
	Actinobacteria	Actinobacteria	Corynebacteriales	Corynebacteriaceae	Corynebacterium 1	50	11	38	46	0,0811	1,92	0,459	0,312	15	39	9,52	19,4
		Actinobacteria	Micrococcales	Micrococcaceae	Kocuria	109	114	17	148	0,00	0,0843	1,65	0,00	0	2,44	4,76	0
		Actinobacteria	Micrococcales	Micrococcaceae	Rothia	15	226	181	152	2,09	0,00056	0,00	0,00	30	2,44	0	0
		Bacteroidia	Bacteroidales	Bacteroidaceae	Bacteroides	9	3	7	7	3,52	**6,29**	2,96	2,85	40	87,8	57,1	64,5
		Bacteroidia	Bacteroidales	Bacteroidales RF16 group	unknown genus	36	13	9	6	0,352	1,70	2,77	3,54	20	68,3	52,4	64,5
		Bacteroidia	Bacteroidales	Porphyromonadaceae	Porphyromonas	133	25	100	167	0,00	0,963	0,0724	0,00	0	24,4	2,38	0
	Bacteroidetes	Bacteroidia	Bacteroidales	Prevotellaceae	Alloprevotella	48	39	28	19	0,0917	0,571	0,736	1,17	10	39	35,7	41,9
		Bacteroidia	Bacteroidales	Prevotellaceae	Prevotellaceae UCG-003	28	20	20	11	0,677	1,17	1,46	1,88	20	65,9	45,2	64,5
		Bacteroidia	Bacteroidales	Rikenellaceae	Alistipes	21	26	22	10	1,44	0,963	1,18	1,94	20	70,7	47,6	51,6
		Bacteroidia	Bacteroidales	Rikenellaceae	Rikenellaceae RC9 gut group	46	17	14	20	0,107	1,42	1,99	1,14	20	70,7	47,6	41,9
		Bacteroidia	Cytophagales	Spirosomaceae	Pseudarcicella	4	14	43	180	**5,01**	1,55	0,403	0,00	15	39	7,14	0
		Bacilli	Bacillales	Bacillaceae	Bacillus	17	88	227	73	1,98	0,143	0,00	0,138	15	17,1	0	6,45
		Bacilli	Bacillales	Staphylococcaceae	Staphylococcus	78	72	54	22	0,00218	0,214	0,304	0,819	5	12,2	4,76	12,9
		Bacilli	Lactobacillales	Streptococcaceae	Streptococcus	2	31	8	5	**9,41**	0,821	2,89	**5,76**	35	43,9	14,3	29
		Clostridia	Clostridiales	Christensenellaceae	Christensenellaceae R-7 group	13	55	32	27	2,15	0,34	0,656	0,744	15	41,5	42,9	51,6
		Clostridia	Clostridiales	Clostridiaceae 1	Multi-affiliation	27	48	34	18	0,91	0,4	0,591	1,22	15	43,9	31	32,3
		Clostridia	Clostridiales	Family XI	Helcococcus	49	12	108	120	0,0907	1,81	0,0614	0,00355	5	43,9	7,14	3,23
		Clostridia	Clostridiales	Family XI	Parvimonas	204	24	144	66	0,00	0,972	0,00423	0,151	0	12,2	2,38	3,23
		Clostridia	Clostridiales	Family XI	Peptoniphilus	59	9	88	233	0,0446	2,94	0,116	0,00	5	31,7	2,38	0
		Clostridia	Clostridiales	Lachnospiraceae	Acetitomaculum	217	46	18	28	0,00	0,43	1,64	0,735	0	48,8	35,7	45,2
		Clostridia	Clostridiales	Lachnospiraceae	Lachnospiraceae NK3A20 group	24	16	10	13	1,10	1,44	2,68	1,53	5	68,3	57,1	32,3
		Clostridia	Clostridiales	Lachnospiraceae	unknown genus	60	23	21	26	0,0381	1,06	1,22	0,764	5	61	40,5	45,2
		Clostridia	Clostridiales	Multi-affiliation	Multi-affiliation	3	6	5	3	**8,31**	4,18	4,60	**8,44**	40	80,5	57,1	74,2
Vaginal		Clostridia	Clostridiales	Peptococcaceae	unknown genus	7	103	69	50	4,65	0,104	0,204	0,255	5	22	16,7	22,6
	Firmicutes	Clostridia	Clostridiales	Peptostreptococcaceae	Multi-affiliation	10	7	3	9	2,90	4,14	**5,31**	2,47	55	73,2	50	54,8
		Clostridia	Clostridiales	Peptostreptococcaceae	Romboutsia	25	34	36	55	1,08	0,694	0,502	0,222	30	39	33,3	22,6
		Clostridia	Clostridiales	Ruminococcaceae	[Eubacterium] coprostanoligenes group	39	15	6	12	0,248	1,51	3,27	1,70	10	63,4	42,9	45,2
		Clostridia	Clostridiales	Ruminococcaceae	Ruminococcaceae NK4A214 group	12	86	39	45	2,27	0,155	0,451	0,318	20	36,6	26,2	35,5
		Clostridia	Clostridiales	Ruminococcaceae	Ruminococcaceae UCG-002	22	126	103	76	1,34	0,0674	0,071	0,12	10	19,5	9,52	19,4
		Clostridia	Clostridiales	Ruminococcaceae	Ruminococcaceae UCG-005	18	2	2	1	1,90	**6,96**	**9,77**	**16,5**	45	78	64,3	83,9
		Clostridia	Clostridiales	Ruminococcaceae	Ruminococcaceae UCG-009	76	51	40	25	0,00487	0,367	0,449	0,77	5	48,8	19	35,5
		Clostridia	Clostridiales	Ruminococcaceae	Ruminococcaceae UCG-010	11	8	15	4	2,40	3,31	1,91	**7,16**	40	80,5	45,2	77,4
		Clostridia	Clostridiales	Ruminococcaceae	Ruminococcaceae UCG-013	82	21	19	14	0,00112	1,16	1,54	1,51	5	56,1	42,9	61,3
		Clostridia	Clostridiales	Ruminococcaceae	Ruminococcaceae UCG-014	74	18	12	21	0,00507	1,30	2,21	1,14	5	58,5	54,8	48,4
		Clostridia	Clostridiales	Ruminococcaceae	Ruminococcus 2	246	45	16	70	0,00	0,455	1,66	0,145	0	46,3	35,7	16,1
		Clostridia	Clostridiales	Ruminococcaceae	unknown genus	19	27	24	15	1,77	0,924	0,994	1,50	10	56,1	42,9	54,8
		Erysipelotrichia	Erysipelotrichales	Erysipelotrichaceae	Turicibacter	43	29	25	23	0,179	0,887	0,832	0,805	25	58,5	38,1	45,2
		Negativicutes	Selenomonadales	Acidaminococcaceae	Phascolarctobacterium	31	50	30	24	0,568	0,374	0,666	0,798	20	46,3	35,7	45,2
		Alphaproteobacteria	Rhodobacterales	Rhodobacteraceae	Paracoccus	40	87	11	53	0,236	0,152	2,24	0,23	5	12,2	2,38	9,68
		Alphaproteobacteria	Sphingomonadales	Sphingomonadaceae	Rhizorhapis	8	301	293	286	3,79	0,00	0,00	0,00	20	0	0	0
		Alphaproteobacteria	Sphingomonadales	Sphingomonadaceae	Sphingomonas	281	19	41	16	0,00	1,18	0,446	1,49	0	29,3	4,76	9,68
	Proteobacteria	Gammaproteobacteria	Aeromonadales	Succinivibrionaceae	Succinivibrio	286	37	23	75	0,00	0,622	1,06	0,125	0	46,3	31	16,1
		Gammaproteobacteria	Betaproteobacteriales	Burkholderiaceae	Polynucleobacter	23	56	50	305	1,18	0,333	0,361	0,00	10	4,88	7,14	0
		Gammaproteobacteria	Pasteurellales	Pasteurellaceae	Histophilus	1	4	1	2	**16,5**	**5,08**	**14**	**11,7**	25	29,3	35,7	29
		Gammaproteobacteria	Pseudomonadales	Moraxellaceae	Acinetobacter	6	40	13	91	4,72	0,539	2,13	0,0728	15	34,1	21,4	3,23
	Tenericutes	Mollicutes	Mycoplasmatales	Mycoplasmataceae	Ureaplasma	16	1	4	17	2,09	**9,45**	4,68	1,26	25	61	31	19,4
	Fusobacteria	Fusobacteriia	Fusobacteriales	Fusobacteriaceae	Fusobacterium	258	5	148	265	0,00	4,22	0,00212	0,00	0	34,1	2,38	0
	Patescibacteria	Parcubacteria	Candidatus Adlerbacteria	unknown family	unknown genus	5	178	53	72	4,93	0,022	0,309	0,142	5	2,44	2,38	3,23
	Verrucomicrobia	Verrucomicrobiae	Verrucomicrobiales	Verrucomicrobiaceae	Prosthecobacter	20	343	31	343	1,60	0,00	0,657	0,00	5	0	4,76	0

						rank(1)				mean abundance (%)				prevalence (%)			
Site	Phylum	Class	Order	Family	Genus	-1W	1M	3M	7M	-1W	1M	3M	7M	-1W	1M	3M	7M
		Actinobacteria	Bifidobacteriales	Bifidobacteriaceae	Bifidobacterium		10	30	81		2,04	0,858	0,161		50,9	24,8	4,96
		Actinobacteria	Corynebacteriales	Corynebacteriaceae	Corynebacterium 1		3	7	6		**6,28**	3,66	3,77		71,3	40,3	42,6
	Actinobacteria	Actinobacteria	Micrococcales	Microbacteriaceae	Multi-affiliation		82	35	5		0,202	0,694	3,93		5,26	14,1	47,5
		Actinobacteria	Micrococcales	Micrococcaceae	Glutamicibacter		58	103	22		0,324	0,108	0,855		18,1	7,38	11,3
		Actinobacteria	Micrococcales	Micrococcaceae	Kocuria		98	63	18		0,161	0,297	1,22		7,02	3,36	14,9
		Bacteroidia	Bacteroidales	Bacteroidaceae	Bacteroides		21	19	34		1,10	1,26	0,541		48,5	26,8	20,6
	Bacteroidetes	Bacteroidia	Flavobacteriales	Flavobacteriaceae	Flavobacterium		24	50	99		0,858	0,474	0,115		35,7	16,1	4,96
		Bacteroidia	Flavobacteriales	Weeksellaceae	Chryseobacterium		19	25	16		1,26	1,07	1,22		32,2	21,5	26,2
		Bacteroidia	Flavobacteriales	Weeksellaceae	unknown genus		31	16	177		0,628	1,40	0,0078		25,7	16,8	0,709
		Bacilli	Bacillales	Bacillaceae	Bacillus		71	96	19		0,242	0,121	1,14		15,2	4,03	23,4
		Bacilli	Bacillales	Planococcaceae	Kurthia		15	8	135		1,51	3,10	0,0468		35,1	39,6	1,42
		Bacilli	Bacillales	Planococcaceae	Multi-affiliation		2	1	20		**7,45**	**7,87**	1,01		69	48,3	24,1
		Bacilli	Bacillales	Staphylococcaceae	Staphylococcus		1	2	1		**8,45**	**7,65**	**13,5**		56,1	30,9	52,5
		Bacilli	Lactobacillales	Aerococcaceae	Aerococcus		20	29	31		1,11	0,862	0,61		42,7	22,8	17,7
		Bacilli	Lactobacillales	Lactobacillaceae	Lactobacillus		14	134	85		1,58	0,0467	0,146		18,7	4,7	2,84
		Bacilli	Lactobacillales	Streptococcaceae	Streptococcus		22	17	24		1,08	1,39	0,773		13,5	9,4	4,96
		Clostridia	Clostridiales	Christensenellaceae	Christensenellaceae R-7 group		42	59	23		0,428	0,382	0,796		33,3	13,4	28,4
		Clostridia	Clostridiales	Clostridiaceae 1	Multi-affiliation		16	23	13		1,50	1,19	2,04		44,4	28,2	26,2
Milk	Firmicutes	Clostridia	Clostridiales	Lachnospiraceae	Acetitomaculum		17	14	15		1,41	1,47	1,47		54,4	27,5	33,3
		Clostridia	Clostridiales	Lachnospiraceae	Lachnospiraceae NK3A20 group		6	3	11		3,97	**6,10**	2,61		69	54,4	47,5
		Clostridia	Clostridiales	Multi-affiliation	Multi-affiliation		4	5	3		**5,43**	4,34	**6,96**		70,8	43,6	61
		Clostridia	Clostridiales	Peptostreptococcaceae	Multi-affiliation		5	9	4		4,59	2,97	3,95		64,9	30,9	49,6
		Clostridia	Clostridiales	Peptostreptococcaceae	Romboutsia		25	48	21		0,835	0,494	0,926		34,5	12,8	24,1
		Clostridia	Clostridiales	Ruminococcaceae	[Eubacterium] coprostanoligenes group		23	24	63		1,05	1,13	0,237		38,6	18,8	9,22
		Clostridia	Clostridiales	Ruminococcaceae	Ruminococcaceae UCG-005		7	4	10		3,76	**5,18**	2,64		64,3	42,3	41,8
		Clostridia	Clostridiales	Ruminococcaceae	Ruminococcaceae UCG-014		18	11	36		1,28	1,77	0,527		53,2	26,8	20,6
		Clostridia	Clostridiales	Ruminococcaceae	Ruminococcus 2		13	18	44		1,63	1,28	0,424		49,7	22,8	12,8
		Erysipelotrichia	Erysipelotrichales	Erysipelotrichaceae	Turicibacter		11	13	17		1,76	1,55	1,22		51,5	28,9	29,8
		Alphaproteobacteria	Rhodobacterales	Rhodobacteraceae	Gemmobacter		102	22	154		0,147	1,19	0,0285		13,5	9,4	1,42
		Alphaproteobacteria	Rhodobacterales	Rhodobacteraceae	Paracoccus		9	10	12		2,07	2,45	2,31		42,1	14,8	32,6
		Alphaproteobacteria	Sphingomonadales	Sphingomonadaceae	Rhizorhapis		12	12	8		1,74	1,61	3,67		36,3	13,4	34,8
		Alphaproteobacteria	Sphingomonadales	Sphingomonadaceae	Sphingomonas		36	15	2		0,492	1,44	**8,54**		15,2	17,4	76,6
	Proteobacteria	Gammaproteobacteria	Betaproteobacteriales	Burkholderiaceae	Ralstonia		38	20	314		0,485	1,22	0,00		2,34	6,04	0
		Gammaproteobacteria	Oceanospirillales	Halomonadaceae	Marinospirillum		158	178	25		0,0541	0,00867	0,772		2,92	0,671	11,3
		Gammaproteobacteria	Pseudomonadales	Moraxellaceae	Acinetobacter		8	6	9		3,55	4,09	2,75		67,3	41,6	39
		Gammaproteobacteria	Pseudomonadales	Moraxellaceae	Psychrobacter		198	26	14		0,022	0,959	1,66		6,43	10,7	31,2
		Gammaproteobacteria	Pseudomonadales	Pseudomonadaceae	Pseudomonas		68	21	7		0,268	1,21	3,76		26,3	32,2	56,7

(1) Ranking was determined for each site and each time point considering the relative abundance of each genus (number of reads corresponding to a given genus compared to the total number of reads for this site and time point)

Only the 25 dominant genera for each site and each time point are listed in Table S2
