# Supplementary material for: Microbiota members from body sites of dairy cows are largely shared within individual hosts throughout lactation but sharing is limited in the herd
Source: Anim Microbiome. 2023 Jun 12;5:32. doi: 10.1186/s42523-023-00252-w (PMC10262541; doi:10.1186/s42523-023-00252-w)
Supplement: Supplementary file 6 — Additional file 6. Dairy cow core microbiota A. Venn diagram representing the core ASVs of the cow nasal, oral, vaginaland milkmicrobiota, 1 week before calvingand 1, 3 and 7 monthspost-partum. Core ASVs were defined for each anatomic site and each time point as ASVs whose relative abundance was higher than 0.01% in at least 50% of samples. A very limited number of core ASV was obtained for each site, in particular vaginal and milk microbiota, with no core ASVs shared between the four anatomic sites. B. For each site and time point, proportion of the core ASVs compared to the total ASV number found in the site at this time point over the 45 cows, and proportion of the core ASVs compared to the number of ASV found in each cow. [file 42523_2023_252_MOESM6_ESM.pdf]

A

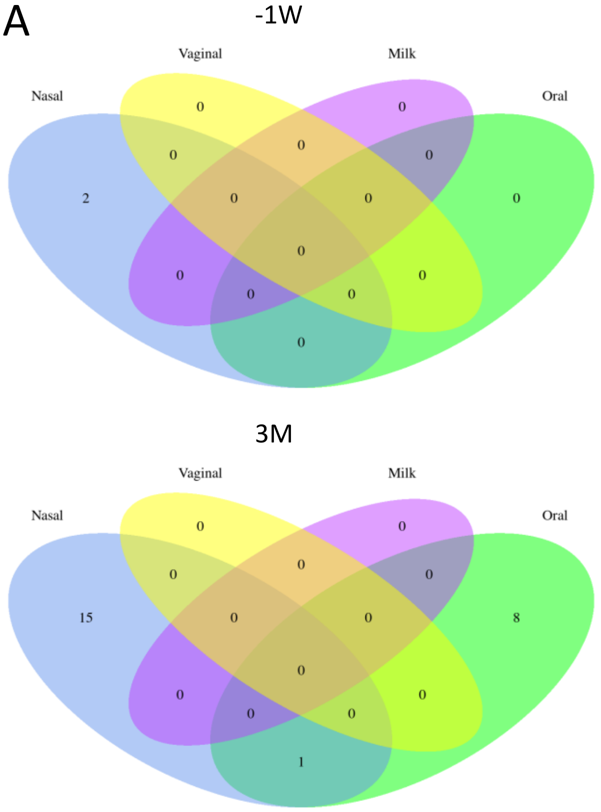

B

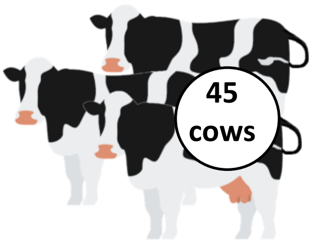

site	time	total number of ASV (1)	number of core ASV (2)	**proportion of core ASV (%)(3)**
O	-1W	136	0	**0.0**
	1M	574	40	**7.0**
	3M	335	9	**2.7**
	7M	334	4	**1.2**
N	-1W	348	2	**0.6**
	1M	837	48	**5.7**
	3M	771	16	**2.1**
	7M	434	7	**1.6**
V	-1W	166	0	**0.0**
	1M	709	6	**0.8**
	3M	542	0	**0.0**
	7M	375	4	**1.1**
M	1M	879	3	**0.3**
	3M	640	0	**0.0**
	7M	592	2	**0.3**

(1) total number of ASV at each site and time point for the 45 cows
(2) number of core ASV at each site and time point for the 45 cows
(3) calculated as follows: (2)/(1)

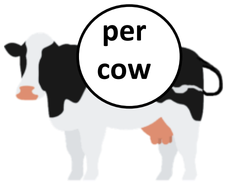

site	time	mean ASV number per sample (1)	**mean percentage of core ASV in each sample (%) (2)**
O	-1W	11	**0.0**
	1M	92	**32.7**
	3M	31	**19.0**
	7M	25	**12.4**
N	-1W	21	**10.0**
	1M	131	**28.5**
	3M	83	**15.1**
	7M	36	**13.4**
V	-1W	14	**0.0**
	1M	84	**7.1**
	3M	48	**0.0**
	7M	46	**6.4**
M	1M	53	**5.4**
	3M	22	**0.0**
	7M	23	**8.6**

(1) mean ASV number per cow for each site at each time point
(2) mean percentage of core ASV per cow for each site at each time point
