# Supplementary material for: Microbiota members from body sites of dairy cows are largely shared within individual hosts throughout lactation but sharing is limited in the herd
Source: Anim Microbiome. 2023 Jun 12;5:32. doi: 10.1186/s42523-023-00252-w (PMC10262541; doi:10.1186/s42523-023-00252-w)
Supplement: Supplementary file 10 — Additional file 10. List of ASVs shared between 3 or 4 anatomic sites in more than 10 animals [file 42523_2023_252_MOESM10_ESM.pdf]

Additional file 10. List of ASVs shared between 3 or 4 anatomic sites in more than 10 animals

ASV ID	number of animals (1)	Phylum	Class	Order	Family	Genus	Species
ASV_29	19	Actinobacteria	Actinobacteria	Bifidobacteriales	Bifidobacteriaceae	Bifidobacterium	Multi-affiliation
ASV_28	14	Actinobacteria	Actinobacteria	Corynebacteriales	Corynebacteriaceae	Corynebacterium 1	Multi-affiliation
ASV_5	18	Firmicutes	Bacilli	Bacillales	Planococcaceae	Multi-affiliation	Multi-affiliation
ASV_4	11	Firmicutes	Bacilli	Lactobacillales	Lactobacillaceae	Lactobacillus	Multi-affiliation
ASV_33	19	Firmicutes	Clostridia	Clostridiales	Clostridiaceae 1	Multi-affiliation	Multi-affiliation
ASV_3	41	Firmicutes	Clostridia	Clostridiales	Multi-affiliation	Multi-affiliation	Multi-affiliation
ASV_47	22	Firmicutes	Clostridia	Clostridiales	Multi-affiliation	Multi-affiliation	Multi-affiliation
ASV_7	37	Firmicutes	Clostridia	Clostridiales	Peptostreptococcaceae	Multi-affiliation	Multi-affiliation
ASV_45	13	Firmicutes	Clostridia	Clostridiales	Peptostreptococcaceae	Multi-affiliation	Multi-affiliation
ASV_31	13	Firmicutes	Clostridia	Clostridiales	Peptostreptococcaceae	Romboutsia	Multi-affiliation
ASV_32	25	Firmicutes	Clostridia	Clostridiales	Ruminococcaceae	Ruminococcaceae UCG-005	unknown species
ASV_26	23	Firmicutes	Erysipelotrichia	Erysipelotrichales	Erysipelotrichaceae	Turicibacter	unknown species
ASV_15	19	Proteobacteria	Alphaproteobacteria	Sphingomonadales	Sphingomonadaceae	Sphingomonas	Multi-affiliation

(1) number of animals for which the ASV was shared between 3 or 4 anatomic sites
